# FGFR3-induced Y158 PARP1 phosphorylation promotes PARP inhibitor resistance via BRG1/MRE11-mediated DNA repair in breast cancer models

**DOI:** 10.1172/JCI173757

**Published:** 2025-05-29

**Authors:** Mei-Kuang Chen, Hirohito Yamaguchi, Yuan Gao, Weiya Xia, Jeffrey T. Chang, Yu-Chun Hsiao, Tewodros W. Shegute, Zong-Shin Lin, Chen-Shiou Wu, Yu-Han Wang, Jennifer K. Litton, Qingqing Ding, Yongkun Wei, Yu-Yi Chu, Funda Meric-Bernstam, Helen Piwnica-Worms, Banu Arun, Jordi Rodon Ahnert, Jinsong Liu, Jun Yao, Wei-Chao Chang, Hung-Ling Wang, Coya Tapia, Constance T. Albarracin, Khandan Keyomarsi, Shao-Chun Wang, Ying-Nai Wang, Gabriel N. Hortobagyi, Chunru Lin, Liuqing Yang, Dihua Yu, Mien-Chie Hung

**Affiliations:** 1 Department of Molecular and Cellular Oncology and; 2 Department of Experimental Radiation Oncology, The University of Texas MD Anderson Cancer Center, Houston, Texas, USA.; 3 Institue of Cell Biology, College of Life Sciences, and; 4 Graduate Institute of Biomedical Sciences, Institute of Biochemistry and Molecular Biology, Research Center for Cancer Biology, Cancer Biology and Precision Therapeutics Center, and Center for Molecular Medicine, China Medical University, Taichung, Taiwan.; 5 Department of General Surgery, Xinhua Hospital Affiliated to Shanghai Jiao Tong University School of Medicine, Shanghai, China.; 6 Department of Integrative Biology and Pharmacology, The University of Texas Health Science Center at Houston, Houston, Texas, USA.; 7 Department of Bioinformatics and Computational Biology,; 8 Department of Breast Medical Oncology, Division of Cancer Medicine,; 9 Department of Anatomic Pathology, Division of Pathology and Laboratory Medicine,; 10 Department of Investigational Cancer Therapeutics,; 11 Department of Breast Surgical Oncology, Division of Surgery,; 12 Sheikh Khalifa Bin Zayed Al Nahyan Institute for Personalized Cancer Therapy, and; 13 Department of Translational Molecular Pathology, The University of Texas MD Anderson Cancer Center, Houston, Texas, USA.

**Keywords:** Oncology, Therapeutics, Breast cancer, Drug therapy, Protein kinases

## Abstract

Poly(ADP-ribose) polymerase (PARP) inhibitors (PARPis) are used to treat *BRCA*-mutated (BRCAm) cancer patients; however, resistance has been observed. Therefore, biomarkers to indicate PARPi resistance and combination therapy to overcome that are urgently needed. We identified a high prevalence of activated FGF receptor 3 (FGFR3) in BRCAm triple-negative breast cancer (TNBC) cells with intrinsic and acquired PARPi resistance. FGFR3 phosphorylated PARP1 at tyrosine 158 (Y158) to recruit BRG1 and prolong chromatin-loaded MRE11, thus promoting homologous recombination (HR) to enhance PARPi resistance. FGFR inhibition prolonged PARP trapping and synergized with PARPi in vitro and in vivo. High-level PARP1 Y158 phosphorylation (p-Y158) positively correlated with PARPi resistance in TNBC patient–derived xenograft models, and in PARPi-resistant TNBC patient tumors. These findings reveal that PARP1 p-Y158 facilitates BRG1-mediated HR to resolve the PARP-DNA complex, and PARP1 p-Y158 may indicate PARPi resistance that can be relieved by combining FGFR inhibitors (FGFRis) with PARPis. In summary, we show that FGFRi restores PARP trapping and PARPi antitumor efficacy in PARPi-resistant breast cancer by decreasing HR through the PARP1 p-Y158/BRG1/MER11 axis, suggesting that PARP1 p-Y158 is a biomarker for PARPi resistance that can be overcome by combining FGFRis with PARPis.

## Abstract 2

FGFR3 activation promotes PARP inhibitor resistance in triple negative breast cancer, which can be reversed by the combination of FGFR inhibitors and PARP inhibitors.

## Graphical abstract

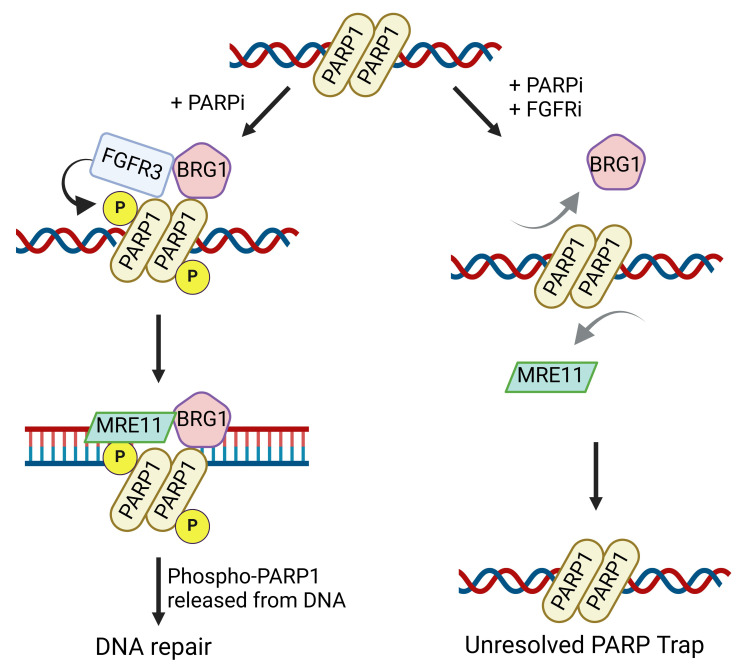

## Introduction

Dissociation of poly(ADP-ribose) polymerase 1 (PARP1) from damaged DNA is a crucial step for the completion of DNA repair (1). Consequently, the retention of PARP1 on DNA induced by PARP inhibitors (PARPis) — a phenomenon known as PARP trapping — is more cytotoxic than the inhibition of PARP1’s poly(ADP-ribosyl)ation (PARylation) enzymatic activity induced by PARPis (2, 3). PARP trapping is resolved only upon the successful completion of DNA repair pathways, predominantly through the homologous recombination (HR) repair mechanism (1). This underscores the importance of PARPis, such as talazoparib and olaparib, both potent inducers of PARP trapping, in the clinical treatment of HR-deficient breast and ovarian cancer caused by *BRCA1/2* mutations (BRCAm) (4).

However, despite their effectiveness, both intrinsic and acquired resistance to PARPis has been observed (1, 5–8). In patients with advanced breast cancer possessing BRCAm, the objective response rate to talazoparib is approximately 60%, with similar rates observed in olaparib-treated patients with metastatic breast cancer (7, 8). These findings suggest that a substantial proportion of BRCAm tumor patients, around 40%, do not derive benefits from single-agent PARPi therapy. Notably, in a cohort of patients with metastatic breast cancer harboring BRCAm, who were treated with either olaparib or platinum, 50% developed resistance mechanisms that reinstated functional HR (6). Similarly, secondary mutations reinstating BRCA1/2 function have been identified in 46% of platinum-resistant or recurrent ovarian cancer with *BRCA* mutations (5). Larger and more comprehensive cohort studies have revealed the presence of *BRCA1* reversion in approximately 30% of patients exhibiting resistance to platinum or PARPi therapy (9, 10). Clinical observations have indicated that tumors carrying near-full-length *BRCA1/2* reversion possess a substantial fitness advantage (10). Although the intricate mechanisms leading to *BRCA* reversion make intercepting its formation challenging, it remains crucial because of the implications for therapy resistance (10). Despite the presence of *BRCA* revertant, tumors in these patients remain resistant to PARPis. Consequently, there is a pressing need for innovative therapeutic strategies to overcome PARPi resistance in this specific subset of patients.

Current strategies aimed at overcoming PARPi resistance involve combining the inhibition of multiple DNA repair pathways with PARP1 inhibition (1–3). Unfortunately, these strategies may also jeopardize normal tissues that rely on these DNA repair mechanisms (4–6, 11). Therefore, the pursuit of novel therapeutic approaches that can surmount PARPi resistance while maintaining a broader therapeutic window between normal and cancerous tissues is of paramount importance. An intriguing avenue lies in the potential of targeting overexpressed receptor tyrosine kinases (RTKs) in cancer cells, as these RTKs contributing to resistance may offer a wider differential expression in normal cells, rendering them less susceptible to such treatment.

While PARP trapping highly contributes to the cytotoxic effects induced by PARPis, the factors that intensify PARP trapping remain incompletely characterized. PARP1 engages with DNA through its zinc finger domains, which are susceptible to posttranslational modifications such as serine/threonine/tyrosine phosphorylation, as predicted by algorithmic analyses (12, 13). In light of this, we hypothesized that kinase-mediated protein phosphorylation might influence PARP trapping. Given the widespread clinical use of inhibitors targeting oncogenic kinases, particularly RTKs, which tend to exhibit a more pronounced therapeutic window between normal and cancer cells (14, 15), we undertook an investigation into the involvement of RTKs in PARP trapping and their potential as agents for overcoming PARPi resistance. Our rationale revolves around the identification of an RTK that could serve as a promising therapeutic candidate. The proposed combination therapy involving both a PARPi and an RTK inhibitor could be more conducive to clinical trial evaluation, utilizing the RTK-phosphorylated substrates as biomarkers to identify potentially responsive patients for mechanism-driven, marker-guided precision medicine. Furthermore, such a clinical trial holds the potential to illuminate the role of posttranslational modifications in PARP trapping and HR, aspects that have not yet been comprehensively explored. The intricate connection among RTKs, PARP trapping, and HR remains a novel area awaiting systematic investigation. To embark on this journey, we prioritized our selection of RTK targets through an unbiased screening process, focusing on activated RTKs exhibiting high prevalence in cells with acquired PARPi resistance.

## Results

### Characterizing a panel of SUM149-derived triple-negative breast cancer cells with PARPi resistance.

**Figure 1:**
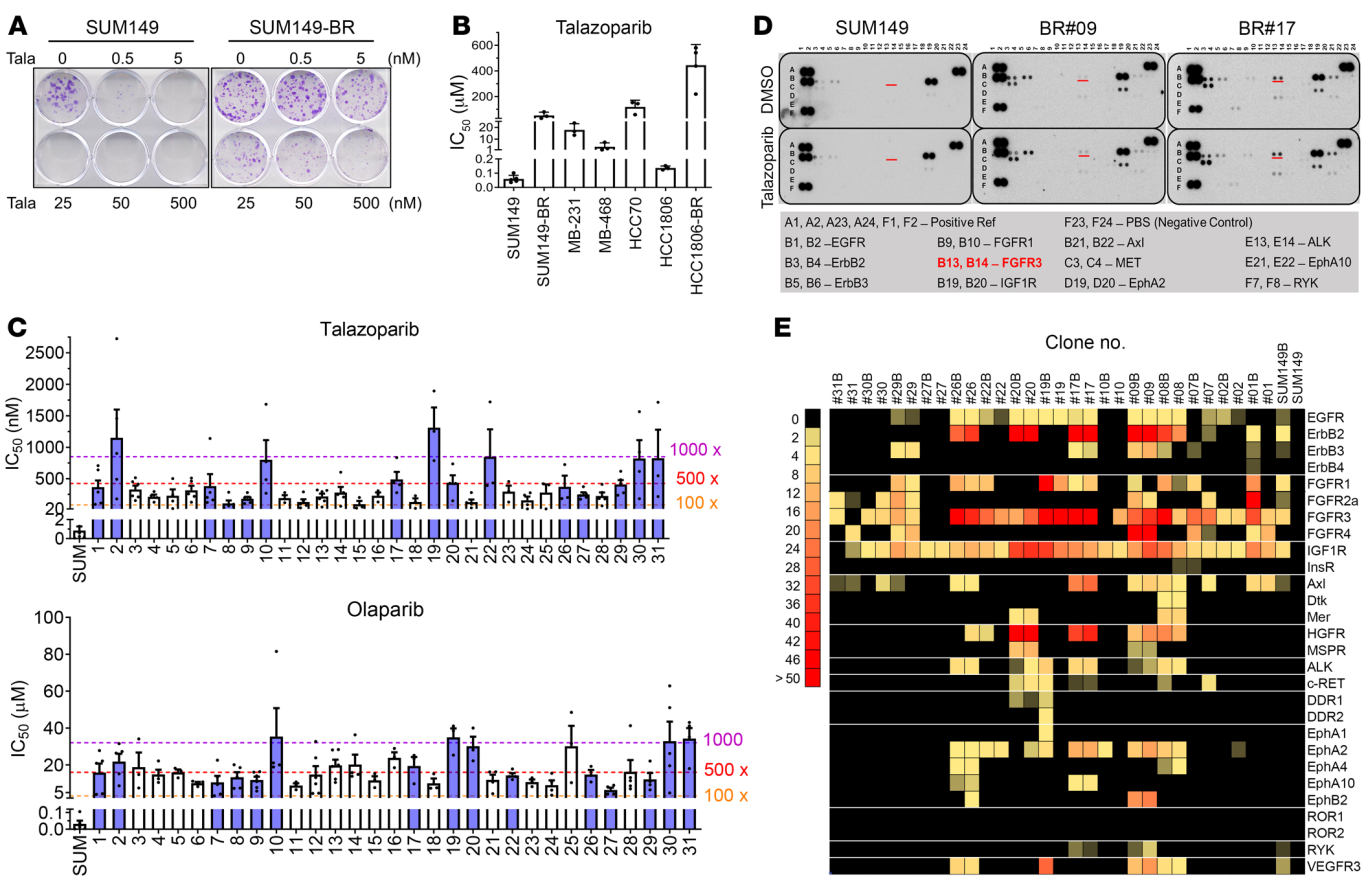
FGFR3 is activated in talazoparib-resistant cells. (**A**) Colony formation of SUM149 parental and BR cells in response to talazoparib. (**B**) Half-maximal inhibitory concentration (IC_50_) of TNBC cells in response to talazoparib. Cells were treated with talazoparib for 4 days before cell survival was analyzed by MTT assay. IC_50_ was calculated using GraphPad Prism 8.0. Histogram shows the mean ± SEM. (Biological repeats: SUM149 *n* = 5, HCC1806BR *n* = 4, all other cell lines *n* = 3.) (**C**) Talazoparib and olaparib IC_50_ of SUM149-BR cells according to MTT assay. Fold change (×) of IC_50_ was compared with that of SUM149 parental cells (SUM). Histogram shows the mean ± SEM (*n* ≥3). The purple bars represent the cells used in the antibody array analysis in **D** and **E**, while the white bars represent the others. (**D** and **E**) Antibody arrays of RTK activation in SUM149 parental and SUM149-BR cells. Cells were treated with DMSO or 100 nM talazoparib overnight and harvested for RTK antibody array analysis. (**D**) The images of RTK antibody arrays in SUM149 parental, BR#09, and BR#17. (**E**) The signal intensities from all the arrays are shown as heatmaps.

We established multiple breast cancer cell lines displaying resistance to PARPis, aiming to identify shared actionable targets contributing to PARPi resistance. To achieve this, we generated talazoparib (BMN673)-resistant cell lines (designated as BR#01 to BR#31) from the initially sensitive *BRCA1*-mutated triple-negative breast cancer (TNBC) cell line SUM149 by exposing them chronically to gradually increasing concentrations of talazoparib (Figure 1A and Supplemental Figure 1A; supplemental material available online with this article; https://doi.org/10.1172/JCI173757DS1). As anticipated, these PARPi-resistant cells exhibited cross-resistance to various PARPis, with a resistance capacity similar to that of the *BRCA1*–wild type TNBC cells (Figure 1B).

The resistance spectrum of these cells to different PARPis was further determined by assessment of the half-maximal inhibitory concentration for each specific PARPi in individual BR cells, revealing a diverse range of responses (Figure 1C and Supplemental Figure 1B). This variance may be due to the involvement of clonal evolution in resistance mechanisms. Additionally, analysis of BRCA1 protein expression unveiled re-expression of BRCA1 in the majority of these BR cell lines (Supplemental Figure 1C), mirroring the clinical observation that *BRCA1* reversion markedly contributes to PARPi and platinum drug resistance (9, 10). Moreover, our prior RNA-Seq data indicated secondary mutations that reinstated the *BRCA1* reading frame in the BR cells, with minimal discrepancies in the expression of *REV7*, *CDK1*, *CHEK1*, and *TP53BP1* between the parental and resistant clones (16).

Conversely, it is also possible that reduced PARP1 protein levels diminish trapping by PARPis, contributing to resistance against these inhibitors. To explore this possibility, we compared PARP1 protein levels in BR cells with those in the parental cells (Supplemental Figure 1D). The data revealed a substantial reduction in PARP1 levels specifically in BR#10 and BR#26 cells. These findings suggest that reduced PARP1 protein may play a role in PARPi resistance in these cells. However, in other resistant cell lines, alternative mechanisms must be contributing to the observed resistance.

### FGF receptor 3 is preferentially activated in PARPi-resistant TNBC cells.

To elucidate the landscape of preferentially activated RTKs with potential for targeted therapies, we assessed a panel of 15 BR cell lines alongside previously published HCC1806 TNBC cells rendered resistant to talazoparib (HCC1806-BR) (17). Using phosphorylated RTK antibody arrays, we aimed to identify RTKs that exhibited higher phosphorylation levels in PARPi-resistant cells compared with their parental counterparts. Quantitative data obtained from the arrays revealed that increased activation of FGF receptor 3 (FGFR3), IGF1R, HGFR/c-MET, ALK, Axl, RYK, and EphA2 was not exclusive to the SUM149-derived BR cells; it was also evident in the independently established HCC1806-BR cells (Figure 1, D and E, and Supplemental Figure 1E). This suggests that heightened phosphorylation of RTKs is a shared feature among TNBC cells exhibiting acquired resistance to PARPis. Among the potential RTK candidates, phosphorylated FGFR3 (p-FGFR3) emerged with the highest prevalence of array signals in SUM149-BR cells, its prevalence being at least 10-fold greater than in the parental cells (Figure 1E).

Given that FGFR3 may contribute to PARPi resistance, we investigated the possibility that *FGFR3*-activating mutations may be involved in PARPi resistance in TNBC. First, we conducted a comprehensive analysis using publicly available cancer datasets. Specifically, we queried the MSK-MET (pan-cancer cohort of 25,775 samples) (18) and MSK-CHORD (breast cancer cohort of 5,368 samples) (19) datasets through cBioPortal (https://www.cbioportal.org/) to identify relevant *FGFR3*-activating mutations. Across these datasets, we identified 12 potential mutations associated with FGFR3 activation, including notable variants R248C, S249C, Y373C, and G380R, which are most prevalent in bladder cancer but occur infrequently in breast cancer (Supplemental Figure 2A). In the breast cancer samples of the MSK-CHORD cohort, we found only 8 *FGFR3*-mutant samples, none of which harbored *BRCA1/2* mutations (Supplemental Figure 2B). Notably, 7 of these samples were hormone receptor^+^/HER2^–^, while one was hormone receptor^–^ and HER2^+^, suggesting an association between *FGFR3* mutations and hormone receptor–positive breast cancer subtypes rather than TNBC. To investigate *FGFR3* mutational status in our PARPi-resistant cell lines, we isolated genomic DNA from 5 resistant clones (BR#2, 7, 9, 17, and 19) and amplified the regions containing the identified *FGFR3* mutation sites by PCR. Sequencing analysis of these regions revealed no activating *FGFR3* mutations in these clones. *FGFR3* gene fusion has also been reported between exons 17 and 18 of the *FGFR3* gene (20), but our sequencing data did not show the *FGFR3* fusion either (data not shown). These findings suggest that *FGFR3* mutations are indeed rare in breast cancer, particularly in TNBC. Therefore, based on both our dataset analysis and sequencing results, we propose that PARPi resistance in our model is more likely driven by FGFR3 upregulation/activation rather than by activating mutations.

We extended our analysis by investigating the correlation between these RTK expressions and breast cancer talazoparib sensitivity, as assessed by the area under the curve (AUC), in the Cancer Dependency Map portal (https://depmap.org/portal/). Among our candidate RTKs, only FGFR3 expression demonstrated a positive correlation trend with talazoparib resistance (Supplemental Figure 3). To delve deeper, we conducted experiments where endogenous FGFR3 was silenced in the BR cell lines, resulting in heightened sensitivity to talazoparib (Supplemental Figure 4A). Conversely, reconstitution of wild-type FGFR3 (FGFR3WT) reinstated resistance to talazoparib (Supplemental Figure 4B). Collectively, these findings suggest that FGFR3 may contribute to PARPi resistance and present a compelling target for overcoming PARPi resistance in breast cancer treatment. As a result, we focused our investigation on the potential of integrating FGFR inhibitors (FGFRis) into PARPi combination therapy and identifying biomarkers that can indicate the presence of FGFR3-mediated PARPi resistance.

### Synergy of FGFRis and PARPis in vitro is independent of full-length BRCA1 expression.

Subsequently, we aimed to determine whether the combination of PARPis and FGFRis could potentially restore sensitivity in PARPi-resistant cells. We employed the Chou-Talalay combination index, where values below 1 indicate synergy (21), to evaluate the synergy between FGFRis and PARPis. To align with potential sponsor interests, we designed treatment combinations that paired talazoparib with PD173074, and olaparib with AZD4547, both originating from the same pharmaceutical company.

**Figure 2:**
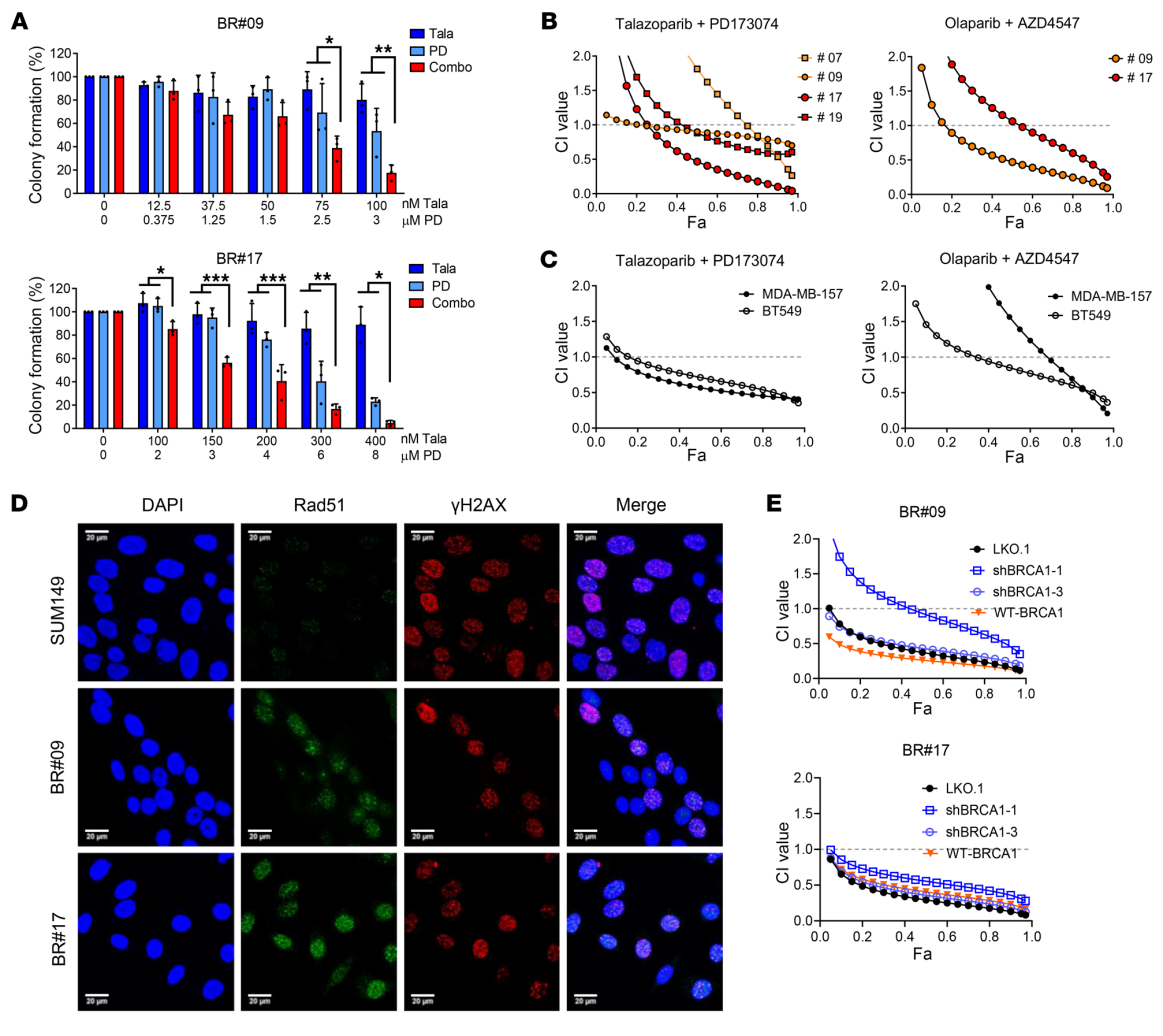
Synergy between PARPis and FGFRis is independent of BRCA1 expression. (**A**) BR#09 and BR#17 cells were treated with talazoparib (Tala) and PD173074 (PD), either alone or in combination (Combo), at the concentrations indicated for 10–12 days, and then cells were fixed for the colony formation assay. The number of colonies formed was normalized to that in the control group (not treated with talazoparib and PD173074), and the mean ± SD from 3 independent experiments is shown in the histogram. *P < 0.05, **P < 0.01, and ***P < 0.001. ANOVA was used for statistical comparisons. Representative images of colony formation are shown in Supplemental Figure 4, C and D. (**B** and **C**) Combination index (CI) of the talazoparib and PD173074 combination or the olaparib and AZD4547 combination in SUM149-BR (**B**), BT549 (**C**), and MDA-MB-157 (**C**) cells. Cells were treated with various concentrations of talazoparib and PD173074 or olaparib and AZD4547 for 4 days before cell survival was measured by MTT assay and the CI was calculated by CompuSyn. Fa, fraction affected. (**D**) Immunofluorescence of SUM149 parental, BR#09, and BR#17 cells staining for DAPI (DNA), RAD51 foci (homologous repair), and γ-H2AX foci (double-strand breaks) after 24 hours of 50 nM talazoparib treatment. Scale bars: 20 μm. (**E**) BRCA1 was knocked down with 2 different shRNAs (shBRCA1-1 and shBRCA1-3) in BR#09 and BR#17 cells. Moreover, BRCA1 was re-expressed in BR#09 and BR#17 shBRCA1-3 cells (WT-BRCA1). These cells, including the control cells (LKO.1), were treated with various concentrations of talazoparib and PD173074 combination, and the CI values were determined. The expression of BRCA1 was determined by Western blot, and the results are shown in Supplemental Figure 4, G and H.

Upon colony formation assays, the talazoparib and PD173074 combination exhibited moderate synergy in BR#09 cells and strong synergy in BR#17 cells (Figure 2A and Supplemental Figure 4, C and D). These clones were mainly used for all later experiments because they exhibited proliferation rates similar to those of the parental cells. However, BR#09 and BR#17 exhibited different drug sensitivity (Figure 2A and Supplemental Figure 4, C and D). There are several possible reasons for the difference in drug sensitivity between BR#09 and BR#17. For example, as shown in the antibody array results in Figure 1D, BR#17 exhibited higher overall RTK activity compared with BR#09, which may contribute to its increased drug resistance. Additionally, BR#17 may have elevated expression of other molecules associated with drug sensitivity, such as ATP-binding cassette transporters, which could enhance its responsiveness to treatment.

Furthermore, we used the MTT assay to assess synergy in both BR cells and intrinsic PARPi-resistant TNBC cells. In BR cells, both combination regimens (talazoparib plus PD173074 and olaparib plus AZD4547) demonstrated moderate to strong synergy (with combination index values ranging between 0.1 and 0.8 when more than 80% of cells were eliminated; Figure 2B). Intriguingly, these synergy patterns were also observed in intrinsically PARPi-resistant BT549 and MDA-MB-157 cells (Figure 2C), two spontaneous TNBC cell lines characterized by increased endogenous FGFR3 phosphorylation (22). This implies that the synergistic effect of these combinations is a widespread phenomenon in TNBC, regardless of whether PARPi resistance is acquired or intrinsic. We also employed the highest-single-agent model and determined that the combination of PARPi and FGFRi ranged from additive to synergistic effects (Supplemental Figure 4, E and F).

Importantly, the synergy observed between FGFRi and PARPi combinations remained despite re-expression of BRCA1 p220 in BR#09 and BR#17 cells (Supplemental Figure 1C) and restoration of Rad51 foci formation within the HR pathway (Figure 2D). The synergy between PARPi and FGFRi persisted even upon BRCA1 knockdown, although 2 knockdown cells showed slightly different sensitivity, which may be due to off-target effects of shRNA (Figure 2E and Supplemental Figure 4, G and H). Nonetheless, these findings indicate that the status of BRCA1 p220 expression is not a critical determinant of the synergy between FGFRis and PARPis in TNBC cell lines. These results collectively suggest that the combination of FGFRis and PARPis could emerge as an effective therapeutic strategy for PARPi-resistant patients with tumors exhibiting *BRCA* reversion.

### Combination of FGFRi and PARPi impedes DNA repair efficiency.

**Figure 3:**
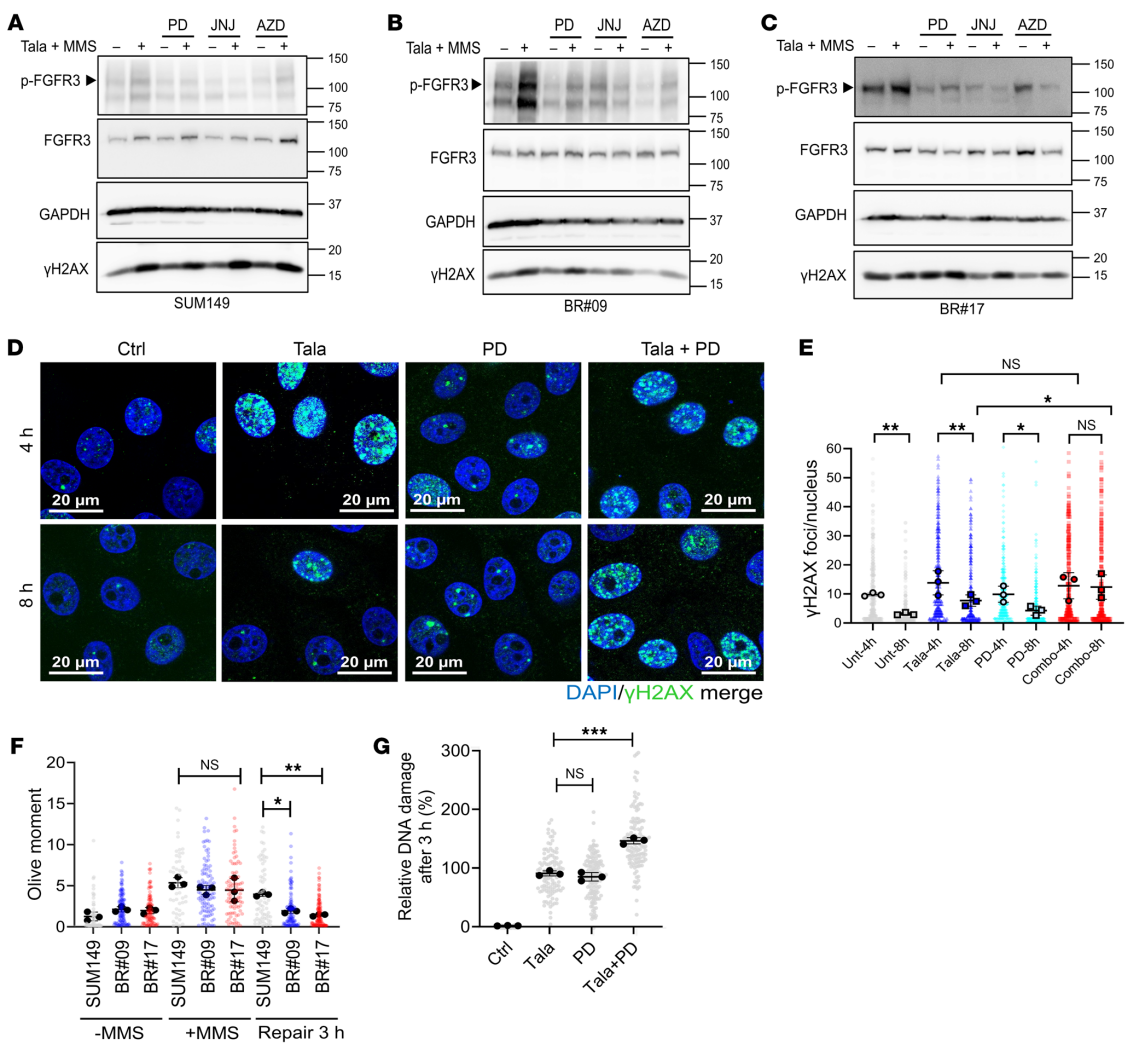
Combination of talazoparib and PD173074 attenuates DNA repair. (**A**–**C**) SUM149 parental (**A**), BR#09 (**B**), and BR#17 (**C**) cells were treated with 5 μM FGFRi (PD173074, JNJ-42756493, AZD4547) for 4 hours, then further exposed for 1 hour to 100 nM talazoparib (Tala) and 0.01% MMS along with the indicated FGFRis before Western blot analysis. (**D** and **E**) BR#17 cells were treated with MMS and the indicated inhibitors for 1 hour, followed by inhibitor treatment after MMS removal. Immunofluorescence images (**D**) display γH2AX (green) and DNA (blue). Scale bars: 20 μm. Scatterplot (**E**) shows mean ± SD from 3 independent experiments; scatterplot represents all counted cells. One-way ANOVA with Tukey’s test: *P < 0.05 and **P < 0.001. (F) The indicated cells were treated with 100 nM talazoparib and 0.01% MMS for 1 hour (+MMS), then recovered in fresh medium for 3 hours before comet assay. Scatterplot displays the mean ± SD from 3 experiments; scatterplot includes all cells counted. One-way ANOVA with Tukey’s test: *P < 0.05 and **P < 0.01. Representative comet assay images are shown in Supplemental Figure 5B. (**G**) BR#09 cells received 0.01% MMS, 100 nM talazoparib, and/or 5 μM PD173074 (alone or combined) for 1 hour before alkaline comet assay. DNA damage (olive moment) was normalized to the talazoparib-treated group. Scatterplot shows mean ± SD from 3 experiments; scatterplot represents all counted cells. One-way ANOVA with Tukey’s test: ***P < 0.001. Representative comet assay images are shown in Supplemental Figure 5C.

To comprehensively understand the involvement of FGFR3 in PARPi resistance, we initiated our exploration by investigating whether FGFR3 activation is a shared aspect of the DNA damage response. We questioned whether DNA alkylating agents, which typically stimulate PARP1 activation, could also trigger phosphorylation of FGFR3. Strikingly, both BR#09 and BR#17 cells exhibited heightened FGFR3 phosphorylation in response to methyl methanesulfonate (MMS) and talazoparib, in comparison with SUM149 parental cells (Figure 3, A–C). Importantly, the phosphorylation of FGFR3 was susceptible to inhibition by FGFRis, such as PD173074, AZD4547, and erdafitinib (Figure 3, A–C). Intriguingly, confocal microscopy imaging demonstrated that FGFR3 colocalized with γH2AX following MMS and talazoparib exposure (Supplemental Figure 5A), implying a potential functional role for FGFR3 in DNA damage repair processes.

We examined the number of γH2AX foci as a marker for DNA double-stranded breaks, often used to gauge the impact of talazoparib treatment. Remarkably, the number of γH2AX foci was comparable between talazoparib-treated cells and cells subjected to the talazoparib and PD173074 combination (Figure 3, D and E). Interestingly, the number of γH2AX foci was substantially reduced after an 8-hour recovery period compared with the 4-hour interval (*P* < 0.001) for cells recovered in inhibitor-free medium, as well as for cells treated and recovered exclusively with talazoparib- or PD173074-containing medium. This pattern indicated DNA repair efficiency within resistant cells in the presence of talazoparib.

However, the DNA repair efficiency was notably compromised in the combination treatment group, as the γH2AX foci count at 8 hours remained similar to that at 4 hours in this group (Combo, Figure 3, D and E). Compared with single-agent treatments, the combination of PARPi and FGFRi hindered the removal of γH2AX DNA breaks, suggesting an impairment in DNA repair efficiency due to the combination treatment. A parallel observation was also noted in comet assay analyses. BR#09 and BR#17 cells exhibited higher efficiency in repairing MMS-induced DNA damage compared with SUM149 parental cells (Figure 3F and Supplemental Figure 5B). The combination of PD173074 and talazoparib in BR#09 cells yielded DNA damage levels akin to those seen with talazoparib alone (repair time, 0 hours; Figure 3G and Supplemental Figure 5C). Strikingly, after 3 hours of MMS removal, most control group cells had eliminated DNA damage. In talazoparib-treated BR#09 cells, the extent of unrepaired DNA damage mirrored that of PD173074-treated cells. Interestingly, the combination of talazoparib and PD173074 resulted in sustained DNA damage levels (repair time, 3 hours; Figure 3G and Supplemental Figure 5C). Moreover, to rule out possible off-target effects of FGFRi, we also confirmed that knockdown of FGFR3 increased DNA damage induced by PARPi (Supplemental Figure 5, D and E).

Findings from the comet assay and γH2AX foci staining collectively indicated that the combination treatment did not initiate more initial DNA damage than talazoparib alone. Instead, the combination of talazoparib and PD173074 notably delayed DNA repair efficiency. This implies that the combination’s augmented cytotoxicity could be attributed to compromised DNA repair efficiency and a sustained DNA break burden.

### FGFR3 phosphorylates PARP1 at tyrosine 158 to enhance PARPi resistance.

**Figure 4:**
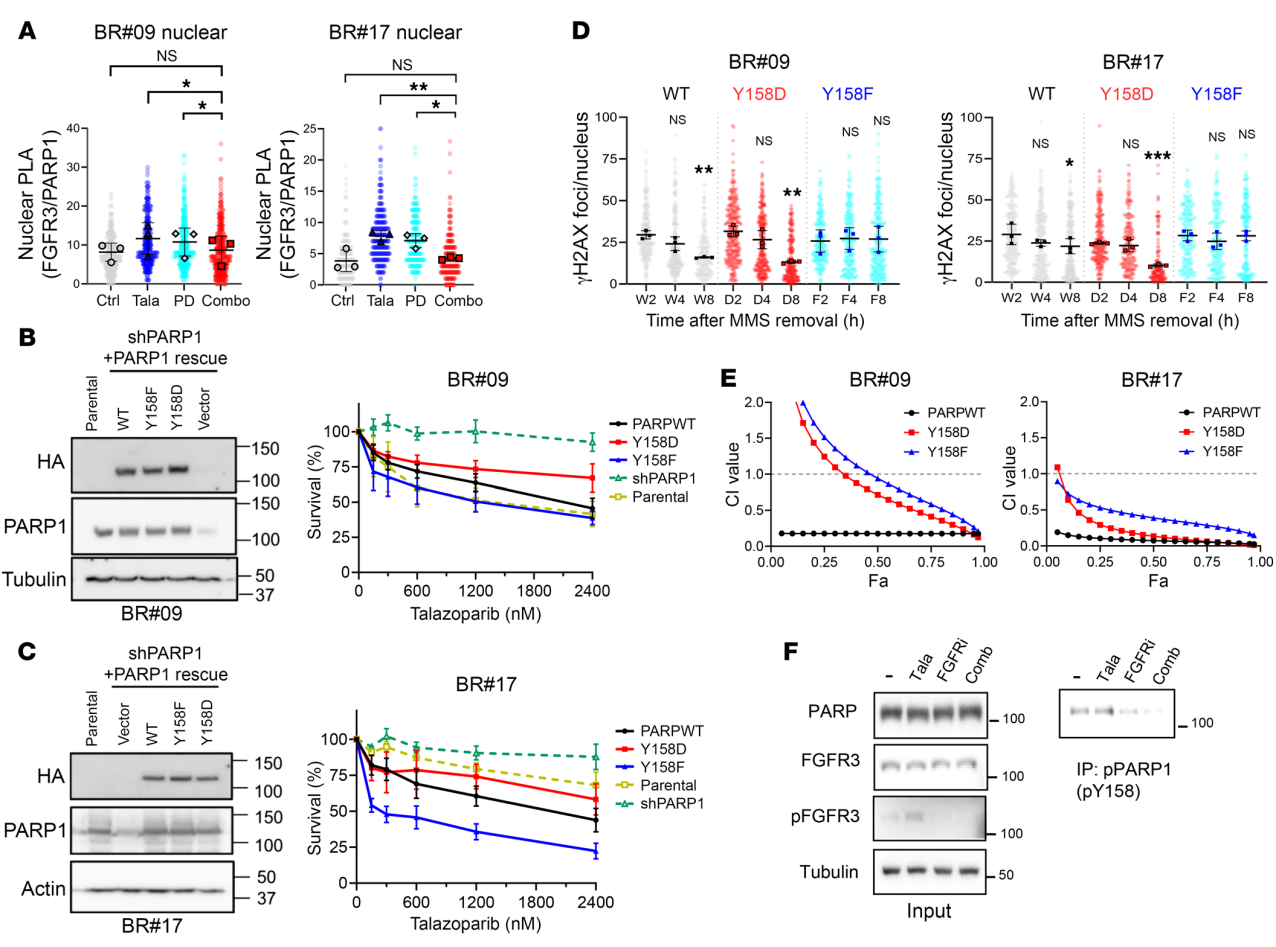
FGFR3 phosphorylates PARP1 at Y158, promoting PARPi resistance. (**A**) BR#09 and BR#17 cells treated with 0.01% MMS and indicated inhibitors (Tala, 100 nM talazoparib; PD, 10 μM PD173074; Combo, Tala+PD) were subjected to PLA using FGFR3 and PARP1 antibodies. Nuclear PLA signals were quantified as mean ± SD from 3 independent experiments; scatterplots show all cells counted. Dunnett’s test: *P < 0.05 and **P < 0.01. Representative images are shown in Supplemental Figure 6, B and C. (**B** and **C**) BR#09 (**B**) and BR#17 (**C**) cells with endogenous PARP1 knockdown (shPARP1) were rescued by exogenous expression of PARP1^WT^, PARP1^Y158D^, or PARP1^Y158F^. PARP1 expression was validated by Western blot (left). Cell survival with talazoparib treatment was assessed by MTT assay (right); mean ± SD from at least 3 independent experiments. (**D**) BR#09 and BR#17 cells expressing PARP1^WT^, PARP1^Y158D^, or PARP1^Y158F^ were treated with MMS (0.01%) and talazoparib (200 nM) for 30 minutes, followed by incubation with 100 nM talazoparib after MMS removal for indicated durations. γH2AX foci were quantified by immunofluorescence (BlobFinder). Scatterplots represent mean ± SD from 3 independent experiments; scatterplots show all cells counted. One-way ANOVA with Tukey’s test was performed to compare time points within each mutant cell line: *P < 0.05; **P < 0.01; ***P < 0.001. Representative images are shown in Supplemental Figure 7C. (**E**) BR#09 and BR#17 cells expressing PARP1^WT^, PARP1^Y158D^, or PARP1^Y158F^ were treated with talazoparib and PD173074 (constant ratio) for 6 days. Cell survival was determined by MTT assay, and combination index (CI) was calculated using CompuSyn. Fa, fraction affected. Results (mean from at least 3 experiments) are shown for BR#09 (left) and BR#17 (right). (**F**) BR#17 cells treated with 0.01% MMS plus 100 nM talazoparib (Tala), 10 μM PD173074 (FGFRi), or their combination were subjected to immunoprecipitation with anti–p-PARP (p-Y158) antibody, followed by Western blotting.

As the combination of FGFRi and PARPi resulted in decreased DNA repair efficiency, we delved into the potential involvement of FGFR3 in mediating PARP1-mediated DNA repair. Notably, coimmunoprecipitation experiments demonstrated the interaction of FGFR3 with PARP1 (Supplemental Figure 6A). Further insights from proximity ligation assays (PLAs) revealed the interaction between FGFR3 and PARP1 within the cellular nucleus (Figure 4A and Supplemental Figure 6, B and C). Notably, the PLA signals were significantly attenuated in cells treated with the combination of talazoparib and PD173074, compared with those treated with either inhibitor alone (Figure 4A and Supplemental Figure 6, B and C). Guided by these findings, we postulated that FGFR3 might potentially serve as a kinase for PARP1. Therefore, we performed in vitro kinase assay using His-tagged PARP1 recombinant protein and active FGFR3 protein, followed by Western blot analysis with antibodies against phosphorylated tyrosine (a mixture of clones 4G10, PY20, and PY100). The result showed obvious tyrosine phosphorylation in PARP1 (Supplemental Figure 6D). Moreover, to identify the specific phosphorylation sites of PARP1, the PARP1 protein phosphorylated by the in vitro kinase assay was analyzed by tandem mass spectrometry (MS/MS). We then identified that PARP1 was phosphorylated at tyrosine 158 (Y158) and 176 (Y176) residues by FGFR3 in vitro (Supplemental Figure 6E).

To further scrutinize the contributions of these phosphorylation events in the context of PARPi resistance, we generated tyrosine-to-phenylalanine (Y to F) mutated PARP1 to simulate unphosphorylatable PARP1, and tyrosine–to–aspartic acid (Y to D) mutated PARP1 to mimic phosphorylated PARP1. MTT assay outcomes revealed that BR cells expressing PARP1^Y158F^ displayed heightened sensitivity to talazoparib, whereas those expressing PARP1^Y158D^ exhibited resistance to talazoparib, when compared with BR cells expressing wild-type PARP1 (PARP1^WT^) (Figure 4, B and C). However, the impact of PARP1^Y176F^ was not considerably pronounced in terms of cell survival in response to talazoparib (Supplemental Figure 7, A and B). Collectively, these results emphasize that FGFR3-mediated phosphorylation of PARP1 at Y158, rather than Y176, emerges as a pivotal determinant in conferring PARPi resistance.

Consistent with the observed delayed removal of γH2AX foci in BR cells exposed to the combination of PARPi and FGFRi, both PARP1^WT^ and PARP1^Y158D^ BR cells exhibited a reduction in the number of γH2AX foci after 8 hours of talazoparib treatment, compared with levels after 2 hours of treatment (*P* < 0.01). In contrast, PARP1^Y158F^ BR cells did not exhibit a decline in the quantity of γH2AX foci (Figure 4D and Supplemental Figure 7C). These findings suggest that both PARP1^WT^ and PARP1^Y158D^ BR cells execute DNA damage repair more effectively than PARP1^Y158F^ BR cells.

The synergy observed between talazoparib and PD173074 was decreased in BR cells carrying either PARP1^Y158F^ or PARP1^Y158D^, when contrasted with PARP1^WT^ cells (Figure 4E). Although the data suggest that the synergism between PARPi and FGFRi may not be solely due to the PARP1 phosphorylation, our results underscore the role of PARP1 Y158 phosphorylation in orchestrating the synergistic effect. Moreover, we detected Y158-phosphorylated PARP1 (p-Y158 PARP1) in BR#17 cells by immunoprecipitation using a specific monoclonal antibody, and notably, the phosphorylation levels were diminished upon treatment with the talazoparib/PD173074 combination (Figure 4F). Taken together, the results suggest that FGFR3 phosphorylates PARP1 at Y158 to enhance PARPi resistance.

### p-Y158 PARP1 enhances BRG1 recruitment and resolves talazoparib-induced PARP trapping.

Next, we investigated how p-Y158 of PARP1 contributes to PARP1 resistance. Since Y158 is located within the DNA-binding zinc finger domain of PARP1, we first examined whether its phosphorylation affects PARP1 enzymatic activity. To do this, we treated PARP1^WT^ and PARP1^Y158F^ BR cells with MMS and analyzed PARylation levels via Western blot using an anti-PAR antibody (Supplemental Figure 8, A and B). The results showed similar PARylation signals between PARP1^WT^ and PARP1^Y158F^ BR cells, suggesting that the PARylation activity of PARP1 remains intact in PARP1^Y158F^ BR cells. These findings indicate that PARPi resistance mediated by PARP1 p-Y158 is not directly linked to changes in PARP1 enzymatic activity.

**Figure 5:**
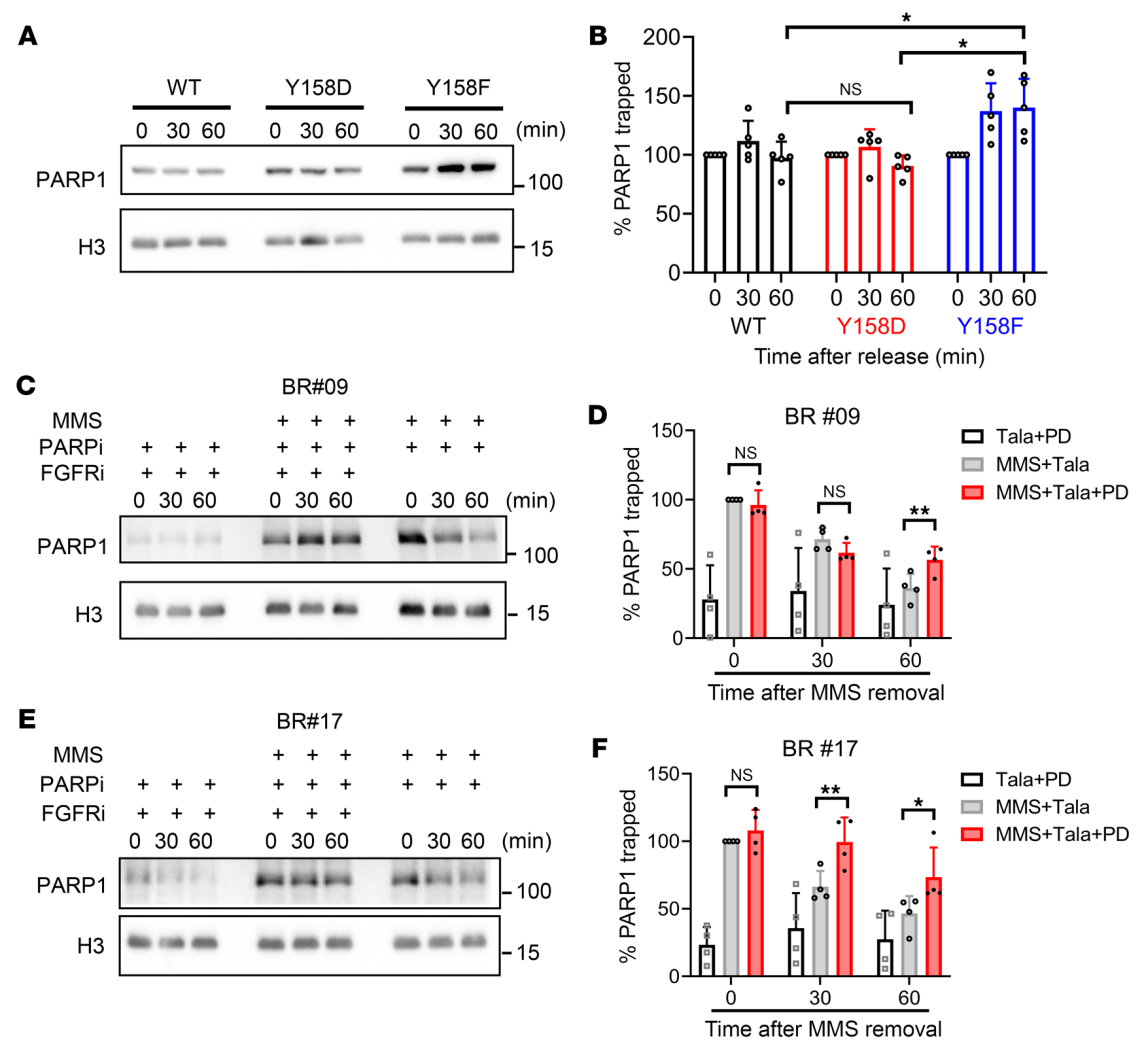
Inhibition of FGFR-mediated PARP1 Y158 phosphorylation prolongs PARP trapping. (**A** and **B**) BR#17 cells expressing PARP1^WT^, PARP1^Y158D^, or PARP1^Y158F^ were harvested at different time points after 40 minutes of treatment with 100 nM talazoparib and 0.01% MMS, and subjected to chromatin fractionation, followed by Western blot (**A**). Chromatin-bound PARP1 signal intensities were normalized to histone H3 and compared with that of the cells at the beginning of DNA repair (0 minutes after releasing from talazoparib and MMS) (**B**). Means ± SD from 5 individual repeats are shown in the histograms. One-way ANOVA was used for statistical comparisons: *P < 0.05. (**C**–**F**) BR#09 and BR#17 cells were pretreated with 10 μM PD173074 for 2 hours, followed by a 40-minute incubation with either PD173074 (10 μM) plus talazoparib (100 nM) plus MMS (0.01%), or PD173074 (10 μM) plus talazoparib (100 nM). Also, these cells were treated with only talazoparib (100 nM) plus MMS (0.01%) for 40 minutes. After drug removal, these cells were harvested at 0, 30, or 60 minutes and subjected to chromatin fractionation. The chromatin-bound PARP1 levels in BR#09 (**C**) and BR#17 (**E**) were then determined by Western blot analysis. PARP1 signal intensities of BR#09 (**D**) and BR#17 (**F**) cells were normalized to histone H3 and compared with that of cells treated with talazoparib and MMS (MMS +, PARPi +, FGFRi +, 0 minutes). Mean ± SD from 4 individual repeats are shown in the histogram. One-way ANOVA with Tukey’s test was used for statistical comparisons: *P < 0.05 and **P < 0.01.

Beyond the inhibition of PARP enzymatic activity, PARPis can exert cytotoxic effects by trapping PARP1 onto damaged DNA (3). With this insight in mind, we extended our examination to the influence of p-Y158 PARP1 on talazoparib-induced chromatin PARP trapping. After permitting cells to repair DNA in the presence of talazoparib following MMS treatment, levels of chromatin-associated PARP1 remained notably elevated in PARP1^Y158F^ BR cells when compared with PARP1^WT^ BR cells (Figure 5, A and B). Conversely, chromatin-associated PARP1 levels in PARP1^Y158D^ BR cells closely resembled those observed in PARP1^WT^ BR cells (Figure 5, A and B). These observations support our hypothesis that p-Y158 PARP1 is less susceptible to talazoparib-induced PARP trapping. This further underscores the role of FGFR3 activation in enhancing PARPi resistance by diminishing PARP trapping. In support of this, we found that PD173074 effectively extended talazoparib-induced PARP1 trapping in BR#09 and BR#17 cells (Figure 5, C–F). Consequently, we concluded that FGFR3-mediated PARPi resistance is brought about by phosphorylation of PARP1 at the Y158 residue, which thereby decreases the extent of PARP trapping resulting from PARPi.

**Figure 6:**
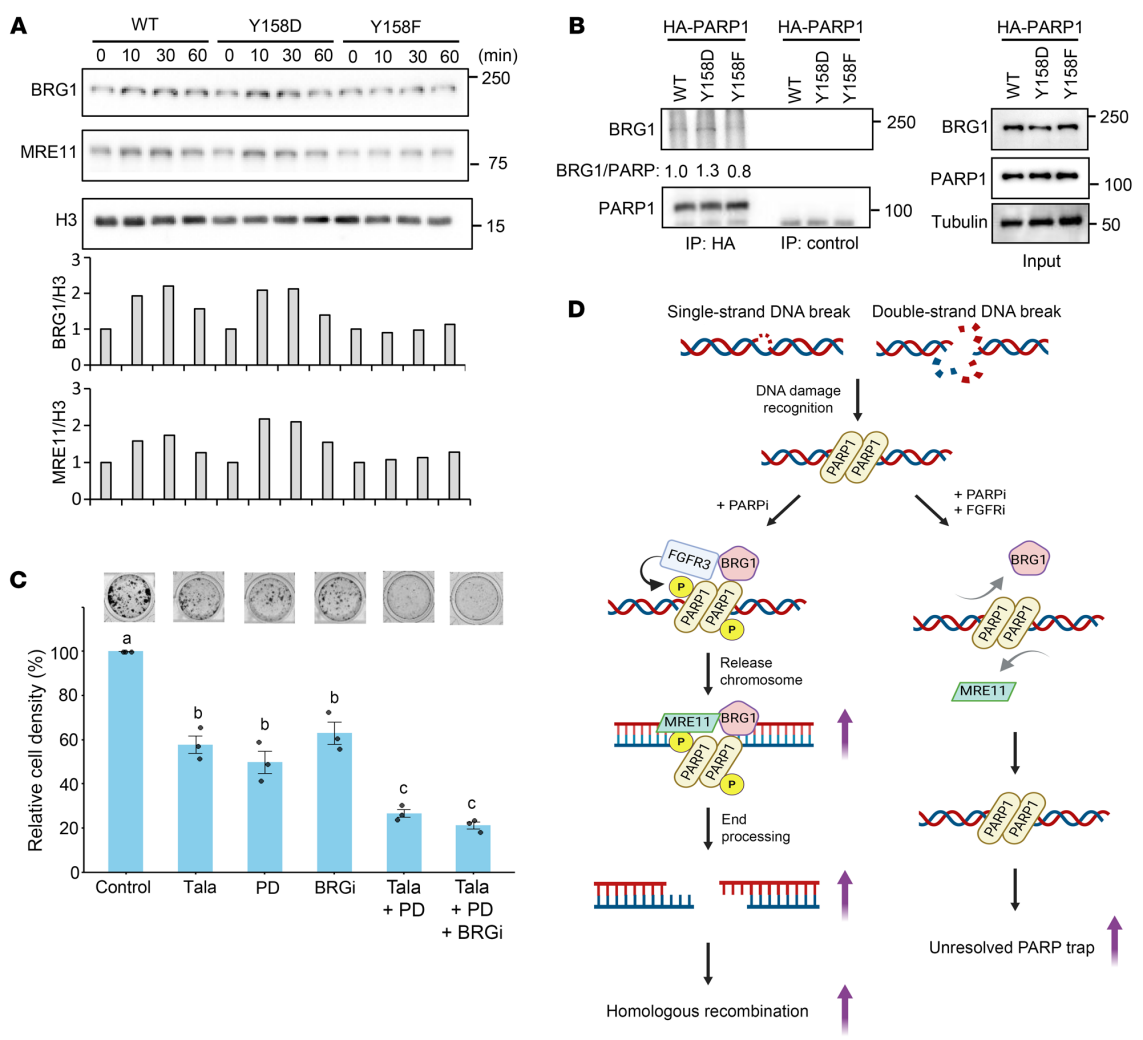
PARP1 Y158 phosphorylation enhances the recruitment of BRG1 and MRE11 on chromatin. (**A**) BR#09 cells expressing PARP1^WT^, PARP1^Y158D^, or PARP1^Y158F^ were treated as described in Figure 5A and harvested at different time points as indicated. Then, chromatin fractions were isolated and subjected to Western blot analysis. Chromatin-bound BRG1 and MRE11 signal intensities were normalized to histone H3 and compared with those of the cells at the beginning of DNA repair (0 minutes after releasing from talazoparib and MMS). Graphs of quantification of BRG1 and MRE11 compared with H3 are shown below. (**B**) BR#09 cells expressing HA-PARP1 wild type or mutants were treated with 100 nM talazoparib and 0.01% MMS for 1 hour and subjected to immunoprecipitation with anti-HA or control IgG, followed by Western blot with the indicated antibodies. (**C**) BR#17 cells were cultured in the presence of 100 nM talazoparib, 2 μM PD173074, 100 nM BRGi, or their combination for 8 days, and cell viability was assessed by colony formation assay. Statistical significance was determined by 1-way ANOVA with Tukey’s test (a vs. b or c, b vs. c: *P* < 0.001). (**D**) The proposed model of FGFR3-mediated PARPi resistance via HR repair regulated by the FGFR3/PARP1/BRG1 complex.

Next, we aimed to elucidate the mechanisms through which FGFR3 contributes to the release of PARP1 trapping. To this end, we used the PANTHER overrepresentation test (http://pantherdb.org/) to analyze the mass spectrum results of FGFR3-interacting proteins in response to talazoparib treatment in SUM149 parental and BR#09 cells. Analyzing the gene ontologies of FGFR3-interacting proteins, we noted that several molecules exhibited enrichment in BR#09 cells but not in SUM149 parental cells. This included proteins involved in nucleosomal DNA binding pathways (Supplemental Table 1). Among the enriched FGFR3-interacting proteins in BR#09 cells, Brahma-related gene 1 (BRG1), a chromatin-remodeling protein known to interact with PARP1 to regulate HR repair (23–25), stood out. This prompted us to explore whether FGFR3 contributes to PARP1-related DNA repair through the regulation of the PARP1-BRG1 interaction. To investigate this, we examined chromatin-bound BRG1 in BR cells expressing PARP1^WT^, PARP1^Y158D^, or PARP1^Y158F^. Intriguingly, both PARP1^WT^ and PARP1^Y158D^ BR cells showed increased levels of chromatin-bound BRG1 during the repair of talazoparib- and MMS-induced DNA damage. In contrast, PARP1^Y158F^ BR cells showed no increase in chromatin-associated BRG1 (Figure 6A and Supplemental Figure 8C). Consistent with this result, the association of PARP1 with BRG1 after PARPi treatment was increased in PARP1^Y158D^ BR cells whereas it was decreased in PARP1^Y158F^ BR cells compared with PARP1^WT^ BR cells (Figure 6B). Notably, a similar trend to that of chromatin-bound BRG1 was observed for chromatin-bound MRE11 protein (Figure 6A and Supplemental Figure 8D), providing further confirmation of the crucial role of p-Y158 PARP1 in upholding HR repair via the BRG1 axis. To further verify the role of BRG1 in FGFR3-mediated PARPi resistance, we investigated the effects of combining PARPi and FGFRi in the presence and absence of a BRG1 inhibitor (BRGi). Our results showed that the combination of PARPi and FGFRi exhibited a significant effect compared with the single treatment, but the addition of a BRGi did not further enhance the effect of the combination (Figure 6C). These findings support the hypothesis that the effects of the PARPi and FGFRi combination are mediated, at least in part, through inhibition of the BRG1-mediated mechanism.

Since MRE11 — a protein known to interact with BRG1 to promote HR DNA repair — was not detected in the mass spectrometry analysis of FGFR3-interacting proteins, we proposed the following model: FGFR3 first phosphorylates PARP1 at Y158, which leads to the recruitment of BRG1. BRG1 then facilitates the subsequent recruitment of MRE11, thereby enhancing HR DNA repair. This process releases PARPi-induced trapping and therefore contributes to PARPi resistance (Figure 6D).

### Combinations of FGFRi and PARPi display tolerable toxicity while inhibiting tumor growth in orthotopic xenograft TNBC models.

**Figure 7:**
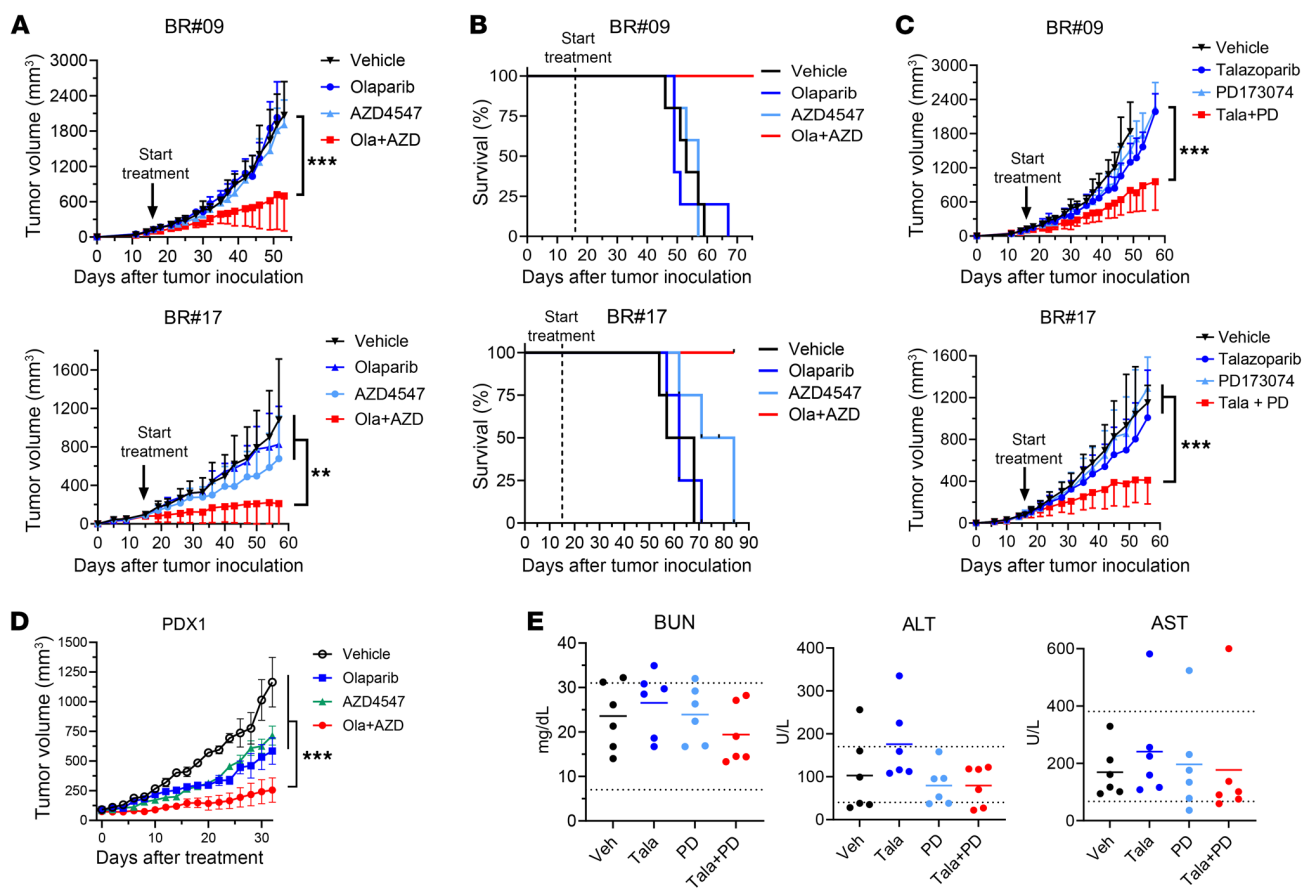
FGFR and PARP inhibitors synergize in breast cancer xenograft models. (**A**) Tumor-bearing mice were treated with olaparib and/or AZD4547. The top graph shows BR#09 xenografts (*n* = 5), and the bottom graph shows BR#17 xenografts (*n* = 4). Tumor volumes were measured over time and analyzed by 1-way ANOVA with Tukey’s test (mean ± SD; **P < 0.01 and ***P < 0.001). (**B**) Survival curves corresponding to the mice in **A**; BR#09 (top) and BR#17 (bottom). (**C**) Tumor growth in BR#09 (*n* = 5) and BR#17 (*n* = 6) xenograft models treated with talazoparib and PD173074, analyzed by 1-way ANOVA with Tukey’s test (mean ± SD; ***P < 0.001). (**D**) The tumor chunks of TNBC PDX model (BCX.070) were inoculated into the fourth mammary fat pad of female nude mice. Once tumors reached 80–100 mm^3^, mice were treated orally with vehicle (*n* = 3), olaparib (*n* = 4), AZD4547 (*n* = 4), or the combination (*n* = 4). Data are presented as the mean ± SEM; 1-way ANOVA with Tukey’s test (***P < 0.001). (**E**) Blood chemical test from 6 BALB/c mice treated with talazoparib and PD173074. Mean values and individual data points are shown. Dotted lines represent the reference levels of alanine aminotransferase (ALT), aspartate aminotransferase (AST), and blood urea nitrogen (BUN) in BALB/c mice.

To ascertain the potential of synergism in an in vivo context, we used xenograft tumor mouse models originating from BR#09 and BR#17 cells, with the aim of evaluating the efficacy of the FGFRi and PARPi combination in curtailing tumor growth. In these models, mice were treated with inhibitor concentrations that mirrored or were lower than the equivalent recommended human doses (26–28). Notably, treatment with olaparib alone exhibited limited tumor growth inhibition, and AZD4547 alone exhibited only marginal growth inhibition in the BR#17 model (*P* = 0.0192 at day 57), while exerting no growth impact on BR#09 tumors (Figure 7A). In stark contrast, the combined administration of olaparib and AZD4547 exerted significant tumor growth inhibition in both models (BR#09, *P* < 0.0001; BR#17, *P* = 0.0046 compared with AZD4547 alone and *P* < 0.0001 compared with vehicle and olaparib alone; Figure 7A). Kaplan-Meier analyses further demonstrated that the combination of olaparib and AZD4547 substantially extended animal survival in both models (Figure 7B).

Similar results were obtained using the talazoparib and PD173074 pair (Figure 7C). First, we had to choose an appropriate dose for PD173074, since it is not for use in clinic so there is no human dose to convert. To this end, we calibrated its concentration for animal use through empirical analysis. Our animal experiments revealed that talazoparib induced FGFR3 phosphorylation, and that PD173074 dose-dependently inhibited talazoparib-induced FGFR phosphorylation in tumor tissues harvested 3 days after treatment (Supplemental Figure 9A). Notably, a subset of mice treated with 20 mg/kg PD173074 per day alongside talazoparib experienced more than 10% weight loss (Supplemental Figure 9B). Consequently, a maximal dose of 15 mg/kg PD173074 per day, devoid of any discernible effect on body weight (Supplemental Figure 9C), was selected for subsequent animal studies. As anticipated, individual administration of talazoparib or PD173074 failed to impede tumor growth in either the BR#09 or the BR#17 model. However, in both models, the combined application of talazoparib and PD173074 exhibited significant tumor growth inhibition (*P* < 0.0001 in both models; Figure 7C), leading to prolonged animal survival, while none of the treated animals experienced weight loss despite the prolonged treatment period (Supplemental Figure 9, C and D). Remarkably, both combinations of PARPi and FGFRi demonstrated the capacity to impede tumor growth and extend animal survival, all while maintaining normal animal body weight in PARPi-resistant xenograft mouse models. We also verified the effect of the combination of PARPi and FGFRi in another resistance TNBC xenograft model using BT549 breast cancer cells as well as a TNBC patient–derived xenograft (PDX) model. PARPi and FGFRi exhibited the synergistic effect on BT549 cells in vitro (Figure 2C). Similarly to the xenograft models using BR cells, the combination of a PARPi and an FGFRi significantly suppressed cancer growth compared with monotherapy in both models (Figure 7D and Supplemental Figure 9E).

Furthermore, we subjected the combination’s toxicity to scrutiny using a syngeneic 4T1 model. Encouragingly, the combination of talazoparib and PD173074 performed better than single-agent treatments in restraining tumor growth over a 2-week treatment regimen before proceeding to toxicity assessments necessitating euthanasia (Supplemental Figure 9F). Notably, levels of blood urea nitrogen, alanine aminotransferase, and aspartate aminotransferase in these animals fell within the normal range for BALB/c mice (Figure 7E), thereby indicating the absence of substantial perturbation to normal kidney or liver function. Moreover, the absence of weight loss over a 50-day treatment period in both models (Supplemental Figure 9, C and G) strongly suggests that the combination holds promise for well-tolerated long-term therapy.

### p-Y158 PARP1 is clinicopathologically relevant in TNBC with PARPi resistance.

**Figure 8:**
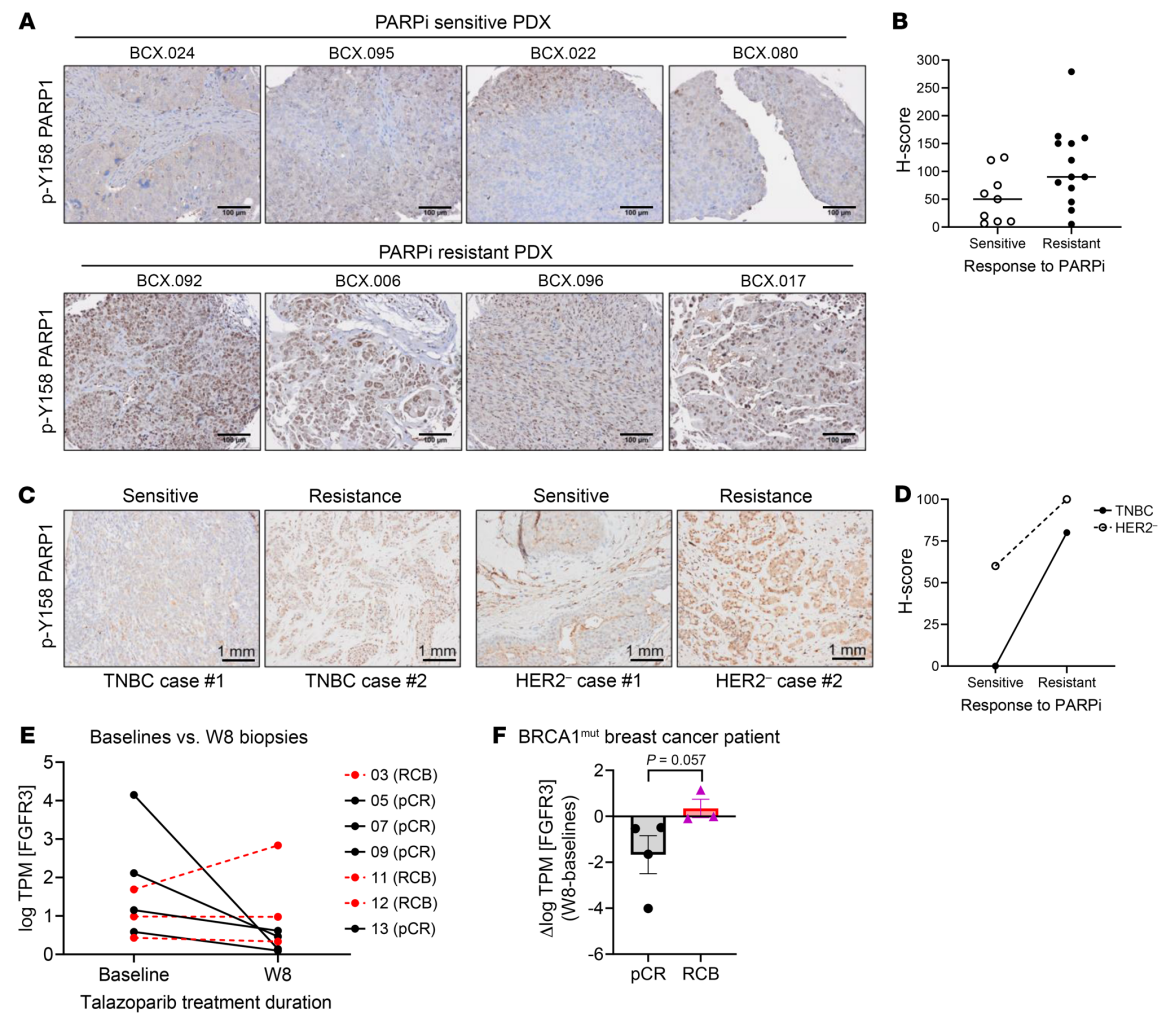
p-Y158 PARP1 is detectable in TNBC patient tumors and PDX models with talazoparib resistance. (**A** and **B**) p-Y158 PARP1 was detected in tissues of TNBC PDX tumors. IHC staining with 3,3′-diaminobenzidine chromogen for monoclonal antibody against p-Y158 PARP1. Representative images and the H-scores of p-Y158 PARP1 are shown in **A** and **B**, respectively (*n* = 9 for sensitive model, *n* = 13 for resistant model). Scale bars: 100 μm. (**C** and **D**) p-Y158 PARP1 was detected in tumor tissues from patients with *BRCA1*-mutated TNBC or HER2^–^ breast cancer with known talazoparib resistance (*n* = 2 each). The images and the H-scores of p-Y158 PARP1 are shown in **C** and **D**, respectively. Scale bars: 1 mm. (**E** and **F**) Baseline (pretreatment) core needle biopsies were collected from treatment-naive patients with *BRCA*-associated breast cancers who consented to receive neoadjuvant talazoparib monotherapy on clinical trial. FGFR3 expression levels (log TPM[FGFR3]) were determined using RNA-seq data from paired biopsies — collected at baseline and at week 8 (W8) of treatment — from a cohort of 7 HER2^–^, *BRCA1*-mutated patients (**E**). Patient treatment outcome was determined by the end of the clinical trial and grouped into pathological complete response (pCR; *n* = 4) and residual tumor burden (RCB; *n* = 3). The *FGFR3* expression level (log TPM[*FGFR3*]) was determined by analysis of RNA-Seq data (**E**). Detailed tumor characterization and RNA-Seq data can be found in a previous publication (32). Mann-Whitney *U* test was used to compare the change of FGFR3 expression level between pCR and RCB patients (**F**).

Given the convergence of in vitro and in vivo evidence substantiating the involvement of p-Y158 PARP1 in PARPi resistance, we undertook the development of a monoclonal antibody against p-Y158 PARP1 for use in tumor immunohistochemistry (IHC) staining. This effort culminated in the successful detection of p-Y158 PARP1 in tumor tissues from TNBC patients (Supplemental Figure 10A). We then conducted an analysis on PDX tumors, derived from individuals who participated in previous clinical trials involving talazoparib for breast cancer treatment (29, 30). Intriguingly, we observed elevated levels of p-Y158 PARP1 in talazoparib-resistant PDX tumors compared with the talazoparib-sensitive tumors (Figure 8, A and B).

To contextualize these findings, we compared the IHC scores for p-Y158 PARP1 and p-FGFR in these PDX models. Notably, we discovered that the p-Y158 PARP1 IHC score exhibited a stronger correlation with PARPi resistance (*P* = 0.03) compared with the p-FGFR IHC score (*P* = 0.66) (Tables 1 and 2 and Supplemental Table 2). Furthermore, by examining PDX models derived from the same patients before and after talazoparib treatment, we discerned higher levels of p-Y158 PARP1 in the post-talazoparib-treatment models relative to their corresponding talazoparib-sensitive models (Supplemental Figure 10B).

Moreover, we extended this investigation to include *BRCA1*-mutated tumors from both TNBC and HER2^–^ breast cancer patients who exhibited PARPi resistance in clinical settings. Importantly, these tumors showed elevated p-Y158 PARP1 levels (Figure 8, C and D). This cumulative evidence led us to conclude that p-Y158 PARP1 holds greater promise as a biomarker for predicting PARPi resistance in TNBC patients compared with p-FGFR, which may detect various p-FGFRs including p-FGFR1, p-FGFR2, and p-FGFR3.

We also analyzed RNA-Seq data from 7 paired *BRCA1*-mutated patient samples (baseline vs. 8 weeks of talazoparib treatment) from the previous clinical trial (7, 31) (Figure 8, E and F). *BRCA* reversion has been found in other cohorts, particularly in 20%–40% of various tumors that progressed from PARPi treatment and/or platinum-based therapy (32–34). However, we did not identify *BRCA1* reversion in this dataset, likely because of the lack of biopsies collected after disease progression from long-term talazoparib treatment. In contrast, our analysis revealed a trend toward increased/maintained *FGFR3* gene expression in biopsies taken during PARPi treatment compared with baseline (pretreatment) biopsies in patients resistant to talazoparib — those who did not achieve complete responses after treatment. Conversely, patients who achieved complete responses exhibited decreased *FGFR3* gene expression in their biopsies during treatment compared with their baseline biopsies. Although the *P* value (0.057) did not reach statistical significance because of the small sample size, and no post-treatment biopsies were available because of the original trial design, this finding provides further support for our hypothesis that sustained *FGFR3* expression contributes to development of PARPi resistance.

## Discussion

The emergence of PARPis as a therapeutic strategy has revolutionized the treatment landscape for *BRCA1/2*-mutated breast and ovarian cancers. However, the development of resistance to these agents poses a significant challenge, necessitating the exploration of novel strategies to overcome resistance mechanisms and improve treatment outcomes. In this study, we unveiled a previously unrecognized role of FGFR3 in mediating PARPi resistance in TNBC. Our comprehensive investigation encompassed both in vitro and in vivo experiments, providing insights into the molecular underpinnings of this resistance mechanism and identifying potential biomarkers for stratifying patient populations for improved therapeutic strategies. The phenomenon of PARP trapping, wherein PARP1 becomes immobilized on damaged DNA, has been established as a major contributor to PARPi-induced cytotoxicity. However, the factors that amplify PARP trapping remained elusive. We hypothesized that kinase-mediated protein phosphorylation could modulate PARP trapping efficiency, and our study illuminated a pivotal role of FGFR3 in this process.

Although FGFR3 is not the only RTK that contributes to PARPi resistance (35–41), it is the first one described that contributes to PARP trapping release. Mechanistically, our study elucidated the role of FGFR3 in mitigating PARP trapping and facilitating DNA repair. We showed that FGFR3 phosphorylates PARP1 at Y158, and that p-Y158 reduces chromatin trapping of PARP1 without affecting its enzymatic activity; in addition, the combination of FGFRi and PARPi prolongs PARP trapping by decreasing BRG1-mediated HR efficiency without increasing the amount of DNA damage. The fact that chromatin-bound BRG1 levels were reduced in cells expressing the unphosphorylatable Y158F mutant of PARP1 underscored the regulatory role of p-Y158 PARP1 in this axis. Notably, our study also unveiled the clinical potential of p-Y158 PARP1 as a biomarker for PARPi resistance. IHC analyses in PDX tumor tissues exhibited elevated p-Y158 PARP1 levels in talazoparib-resistant tumors. This observation suggests that p-Y158 PARP1 holds promise as a predictive biomarker for identifying patients who may not benefit optimally from PARPi monotherapy, thereby facilitating personalized treatment strategies.

Given the results of the current study, several factors are worthy of future investigation. Structurally, the Y158 amino acid is adjacent to the PARP1 zinc finger 2 domain’s zinc ion binding residues (C125, C128, H159, and C162) and DNA-interacting residues (L151/I156) (42, 43), indicating that Y158 may also stabilize the protein tertiary structure (Supplemental Figure 10C). In addition, previous studies showed that PARP1 contributes to double-strand DNA break repair by regulating nucleosome density, and that PARylated chromatin recruits BRG1 to DNA damage sites (24). However, we found that PARP1^Y158F^ has PARylation activity similar to that of PARP1^WT^, but with less capability to retain BRG1 onto DNA (Figure 6 and Supplemental Figure 8). Our results suggest that PARP1 not only contributes to initial recruitment of BRG1 through PAR but also facilitates HR by retaining BRG1 at DNA.

Limitations of the current study primarily arise from the structural conservation among FGFR family members; FGFR1–3 may not contribute to PARPi resistance equally. Because the autophosphorylation sites on FGFR1–3 are highly conserved (44), the antibodies recognizing p-FGFR can cross-react with FGFR1–3, and thus the p-FGFR signal cannot reflect the quantity of p-FGFR3 accurately in Western blot analysis and IHC staining. Using CRISPR/Cas9 knockout screening and drugZ analysis (45), we found that knocking out FGFR2, 3, or 4 sensitized BR cells to talazoparib (drugZ score < 1), whereas knocking out FGFR1 did not induce PARPi sensitization in the BR cells tested (Supplemental Table 3). Although we cannot rule out the possibility that PARP1 Y158 residue can be phosphorylated by other FGFR members in various cancer types, we found that Y158 phosphorylation is predicted to be mediated only by FGFR3 among the FGFR family using the algorithm of group-based phosphorylation site predicting analysis (http://gps.biocuckoo.cn/) (13). Therefore, p-Y158 PARP1 could be a better biomarker in predicting FGFR3-mediated PARPi resistance than p-FGFR in breast cancer tissues.

PLA and coimmunoprecipitation of PARP1 and FGFR3 showed their interaction even in untreated cells (Figure 4A and Supplemental Figure 6, A–C), indicating baseline FGFR3-PARP1 interaction in the nucleus under physiological conditions. Although we did not test multiple cell lines, this interaction was also observed in parental SUM149 cells (Supplemental Figure 6A), and nuclear p-FGFR signal was also detected in PDX tumors (Supplemental Figure 10B), supporting its broad relevance. However, p-Y158 PARP1 IHC score correlated more strongly with PARPi resistance (*P* = 0.03) than p-FGFR IHC score (*P* = 0.66) (Tables 1 and 2 and Supplemental Table 2), suggesting that PARP1 Y158 phosphorylation, rather than FGFR3-PARP1 interaction, may be more specifically associated with FGFR3-mediated PARPi resistance.

Moreover, PARPis primarily induce double-strand breaks during S phase (46), while FGFRis are known to cause G_0_/G_1_ cell cycle arrest. Therefore, FGFRi-induced G_0_/G_1_ arrest may interfere with the effects of PARPis in certain cancer cell populations. Given this, a sequential treatment strategy — administering PARPi first, followed by FGFRi — may be more effective. This hypothesis warrants further investigation in future studies.

In conclusion, our study has unveiled a previously unrecognized role of FGFR3 in mediating PARPi resistance in TNBC through the phosphorylation of PARP1 at Y158. This resistance mechanism involves a reduction in PARP trapping efficiency and altered DNA repair dynamics. The synergistic potential of FGFRi and PARPi combination therapy, as evidenced in both in vitro and in vivo models, presents an exciting avenue for overcoming PARPi resistance. Furthermore, the identification of p-Y158 PARP1 as a biomarker holds significant promise for guiding treatment decisions and optimizing therapeutic outcomes for TNBC patients. This study not only deepens our understanding of PARPi resistance mechanisms but also provides a foundation for the development of innovative treatment strategies in the realm of precision oncology.

## Methods

Further information can be found in Supplemental Methods.

### Sex as a biological variable.

Our study exclusively examined female mice since the disease modeled is mainly relevant in females.

### Y158-phosphorylated PARP1 antibody generation.

Mouse anti–phospho–PARP1 Y158 antisera were generated by immunization of 20 mice with phospho–PARP1 Y158 KLH hot peptide (KLH-C-EKPQLGMIDRW-pY-HPG-S-FVKNREE) once every 2 weeks. Binding affinity specificities of the antisera were evaluated by enzyme-linked immunosorbent assay and Western dot blot with hot peptide (C-EKPQLGMIDRW-pY-HPG-S-FVKNREE) and cold peptide (C-EKPQLGMIDRW-Y-HPG-S-FVKNREE). From the monoclonal antibodies developed, clone 2A1.3 (IgG) was used for immunoprecipitation, and clone 2G9.2 (IgM, 1:50) was used for IHC analysis.

### Tissue samples and IHC staining.

The tissues for generating breast cancer patient–derived xenograft models were collected under MD Anderson Cancer Center Institutional Review Board–approved protocols. Clinical information of BCX models was listed in a previous publication (29). The human breast cancer tissue samples were collected according to University of Texas MD Anderson Cancer Center Institutional Review Board guidelines (protocols ABT-888 phase 2 AbbVie and 2014-0045 Talazoparib Neoadjuvant). All samples were from female patients with breast cancer. TNBC case 1 (ER^–^, PR^–^, HER2^–^) received PARPi single-agent treatment; TNBC case 2 (ER^–^, PR^–^, HER2^–^) was treated with PARPi and received platinum for metastatic disease on trial. HER2^–^ case 1 (ER^+^, PR^+^, HER2^–^) received PARPi single-agent treatment; HER2^–^ case 2 (ER^+^, PR^+^, HER2^–^) received PARPi single-agent treatment. Paraffin-embedded slides were immersed in Histo-Clear (National Diagnostics, catalog HS-200) followed by ethanol for deparaffinization. Heat-induced epitope retrieval was performed with Tris buffer (pH 9.0). Slides were blocked with 3% hydrogen peroxide, BLOXALL blocking solution (Vector Laboratories, catalog SP-6000), and normal goat serum before being incubated with primary antibody against p-Y158 PARP1 (clone 2G9.2, 1:50) or p-FGFR (Biorbyt, catalog orb156865; 1:25). Biotinylated goat anti-mouse IgM antibody (Vector Laboratories, catalog BA-2020) and ABC-HRP kit (Vector Laboratories, catalog PK-6100) were used for signal amplification. Zeiss Zen software was used with a Zeiss LSM 710 confocal microscope for immunofluorescence staining imaging. Zeiss AxioVision was used with a Zeiss light microscope for comet assay imaging. Celigo version 5.0 was used with a Nexcelom Celigo Imaging Cytometer for colony formation imaging and analyses.

### Statistics.

For Western blot signal quantification, signal intensities were analyzed using Image Studio Lite (version 5.2, LI-COR Biosciences). Signals of PARP1, PARylation, FGFR3, and p-FGFR were first normalized to housekeeping proteins (tubulin, actin, or GAPDH) of each sample before being normalized to the control groups. Every independent experiment repeat was quantified individually. Fold changes in Western blot signals were analyzed by a nonparametric Friedman test using GraphPad Prism 8.0 software. *P* values less than 0.05 were considered statistically significant: *P < 0.05, **P < 0.01, and ***P < 0.001. Combination index experiments were designed according to the Chou-Talalay method (47), and results were calculated using CompuSyn software (http://www.combosyn.com/). Pivot table analysis was performed using Microsoft Excel and the *P* value was assessed using the χ^2^ test.

### Study approval.

Animal studies were performed following a protocol (no. 00001250) approved by The University of Texas MD Anderson Cancer Center Institutional Animal Care and Use Committee. The patient-derived xenograft mouse models were generated under MD Anderson Cancer Center Institutional Review Board–approved protocols (LAB07-0950, principal investigator Funda Meric-Bernstam) as stated in the previous study introducing these models (29).

### Code availability.

BlobFinder (48) and CompuSyn (http://www.combosyn.com/) open-source code was used for analyses performed in the current study. The following commercial code was also used for data analyses: Image Studio Lite (https://www.licor.com/bio/image-studio-lite/), Aperio ImageScope (https://www.leicabiosystems.com/digital-pathology/manage/aperio-imagescope/), GraphPad Prism 8, and TriTek CometScore freeware v1.5.

### Data availability.

Values for data points in figures are reported in the Supporting Data Values file. All the datasets generated and/or analyzed during the current study are available on reasonable request.

## Author contributions

MKC designed the project, performed experiments, analyzed data, and wrote the paper with input from all authors. HY, YG, YCH, TWS, ZSL, CSW, YHW, and YYC designed and performed experiments and analyzed data. MKC, WX, YW, and QD performed immunohistochemistry staining and data analysis. JKL, FMB, HPW, and JRA provided insights regarding potential clinical applications. HLW and SCW generated and validated antibodies against the p-PARP1. WCC performed and analyzed mass spectrometry experiments. BA and CTA provided patient tumor tissues. JKL, FMB, HPW, JL, and CT provided and validated patient-derived xenograft models and their tissues. KK provided PARPi-resistant HCC1806 cells. JTC and JY analyzed results from PANTER and cBioportal databases. HY, YNW, GNH, CL, and LY commented on and edited the manuscript. DY and MCH supervised the entire project and wrote the manuscript.

## Supplementary Material

Supplemental data

Unedited blot and gel images

Supporting data values

## Figures and Tables

**Table 1:**
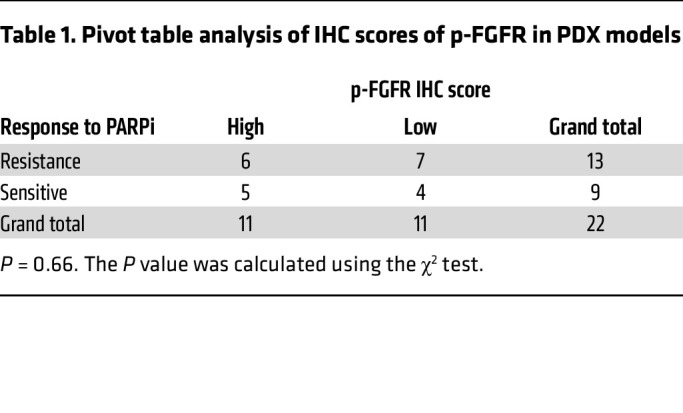
Pivot table analysis of IHC scores of p-FGFR in PDX models

**Table 2:**
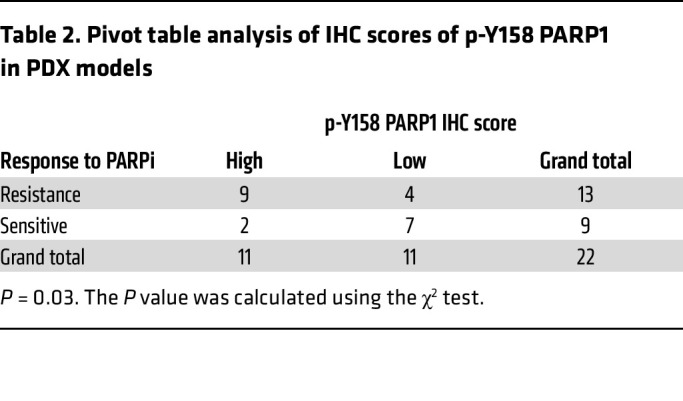
Pivot table analysis of IHC scores of p-Y158 PARP1 in PDX models

## Version 1 06/03/2025

In-Press Preview

## Version 2 07/15/2025

Electronic publication

## References

[1] Li H (2020). PARP inhibitor resistance: the underlying mechanisms and clinical implications. Mol Cancer.

[2] Pommier Y (2016). Laying a trap to kill cancer cells: PARP inhibitors and their mechanisms of action. Sci Transl Med.

[3] Murai J, Pommier Y (2019). Aberrant RNA splicing in cancer. Annu Rev Cancer Biol.

[4] Pilie PG (2019). State-of-the-art strategies for targeting the DNA damage response in cancer. Nat Rev Clin Oncol.

[5] Norquist B (2011). Secondary somatic mutations restoring BRCA1/2 predict chemotherapy resistance in hereditary ovarian carcinomas. J Clin Oncol.

[6] Waks AG (2020). Reversion and non-reversion mechanisms of resistance to PARP inhibitor or platinum chemotherapy in BRCA1/2-mutant metastatic breast cancer. Ann Oncol.

[7] Litton JK (2018). Talazoparib in patients with advanced breast cancer and a germline BRCA mutation. N Engl J Med.

[8] Robson M (2017). Olaparib for metastatic breast cancer in patients with a germline BRCA mutation. N Engl J Med.

[9] Tobalina L (2021). A meta-analysis of reversion mutations in BRCA genes identifies signatures of DNA end-joining repair mechanisms driving therapy resistance. Ann Oncol.

[10] Pettitt SJ (2020). Clinical *BRCA1/2* reversion analysis identifies hotspot mutations and predicted neoantigens associated with therapy resistance. Cancer Discov.

[11] Fang Y (2019). Sequential therapy with PARP and WEE1 inhibitors minimizes toxicity while maintaining efficacy. Cancer Cell.

[12] Gagne JP (2009). Proteomic investigation of phosphorylation sites in poly(ADP-ribose) polymerase-1 and poly(ADP-ribose) glycohydrolase. J Proteome Res.

[13] Wang C (2020). GPS 5.0: an update on the prediction of kinase-specific phosphorylation sites in proteins. Genomics Proteomics Bioinformatics.

[14] Bhullar KS (2018). Kinase-targeted cancer therapies: progress, challenges and future directions. Mol Cancer.

[15] Sharma SV, Settleman J (2007). Oncogene addiction: setting the stage for molecularly targeted cancer therapy. Genes Dev.

[16] Shih DJH (2022). Exploiting induced vulnerability to overcome PARPi resistance and clonal heterogeneity in BRCA mutant triple-negative inflammatory breast cancer. Am J Cancer Res.

[17] Carey JPW (2018). Synthetic lethality of PARP inhibitors in combination with MYC blockade is independent of BRCA status in triple-negative breast cancer. Cancer Res.

[18] Nguyen B (2022). Genomic characterization of metastatic patterns from prospective clinical sequencing of 25,000 patients. Cell.

[19] Jee J (2024). Automated real-world data integration improves cancer outcome prediction. Nature.

[20] Capelletti M (2014). Identification of recurrent FGFR3-TACC3 fusion oncogenes from lung adenocarcinoma. Clin Cancer Res.

[21] Chou TC (2010). Drug combination studies and their synergy quantification using the Chou-Talalay method. Cancer Res.

[22] Smith SE (2017). Molecular characterization of breast cancer cell lines through multiple omic approaches. Breast Cancer Res.

[23] Qi W (2015). BRG1 promotes the repair of DNA double-strand breaks by facilitating the replacement of RPA with RAD51. J Cell Sci.

[24] Chen Y (2019). A PARP1-BRG1-SIRT1 axis promotes HR repair by reducing nucleosome density at DNA damage sites. Nucleic Acids Res.

[25] Moison C (2021). Zinc finger protein E4F1 cooperates with PARP-1 and BRG1 to promote DNA double-strand break repair. Proc Natl Acad Sci U S A.

[26] Nair AB, Jacob S (2016). A simple practice guide for dose conversion between animals and human. J Basic Clin Pharm.

[27] de Bono J (2017). Phase I, dose-escalation, two-part trial of the PARP inhibitor talazoparib in patients with advanced germline *BRCA1/2* mutations and selected sporadic cancers. Cancer Discov.

[28] Paik PK (2017). A Phase Ib open-label multicenter study of AZD4547 in patients with advanced squamous cell lung cancers. Clin Cancer Res.

[29] Evans KW (2017). A population of heterogeneous breast cancer patient-derived xenografts demonstrate broad activity of PARP inhibitor in BRCA1/2 wild-type tumors. Clin Cancer Res.

[30] Litton JK (2020). Neoadjuvant talazoparib for patients with operable breast cancer with a germline *BRCA* pathogenic variant. J Clin Oncol.

[31] Liu X (2022). Identification of biomarkers of response to preoperative talazoparib monotherapy in treatment naïve gBRCA+ breast cancers. NPJ Breast Cancer.

[32] Kondrashova O (2017). Secondary somatic mutations restoring *RAD51C* and *RAD51D* associated with acquired resistance to the PARP inhibitor rucaparib in high-grade ovarian carcinoma. Cancer Discov.

[33] Goodall J (2017). Circulating cell-free DNA to guide prostate cancer treatment with PARP inhibition. Cancer Discov.

[34] Weigelt B (2017). Diverse *BRCA1* and *BRCA2* reversion mutations in circulating cell-free DNA of therapy-resistant breast or ovarian cancer. Clin Cancer Res.

[35] Nowsheen S (2012). Synthetic lethal interactions between EGFR and PARP inhibition in human triple negative breast cancer cells. PLoS One.

[36] Balaji K (2017). AXL inhibition suppresses the DNA damage response and sensitizes cells to PARP inhibition in multiple cancers. Mol Cancer Res.

[37] Xie Y (2019). Systematic analysis of NLMP suggests nuclear localization of RTK/MET kinases resemble cancer cell clearance. J Exp Clin Cancer Res.

[38] Dong Q (2019). EGFR and c-MET cooperate to enhance resistance to PARP inhibitors in hepatocellular carcinoma. Cancer Res.

[39] Chu YY (2022). Targeting the ALK-CDK9-Tyr19 kinase cascade sensitizes ovarian and breast tumors to PARP inhibition via destabilization of the P-TEFb complex. Nat Cancer.

[40] Chu YY (2020). Blocking c-Met and EGFR reverses acquired resistance of PARP inhibitors in triple-negative breast cancer. Am J Cancer Res.

[41] Du Y (2016). Blocking c-Met-mediated PARP1 phosphorylation enhances anti-tumor effects of PARP inhibitors. Nat Med.

[42] Langelier MF (2011). Crystal structures of poly(ADP-ribose) polymerase-1 (PARP-1) zinc fingers bound to DNA: structural and functional insights into DNA-dependent PARP-1 activity. J Biol Chem.

[43] Eustermann S (2011). The DNA-binding domain of human PARP-1 interacts with DNA single-strand breaks as a monomer through its second zinc finger. J Mol Biol.

[44] Hart KC (2001). Identification of tyrosine residues in constitutively activated fibroblast growth factor receptor 3 involved in mitogenesis, Stat activation, and phosphatidylinositol 3-kinase activation. Mol Biol Cell.

[45] Colic M (2019). Identifying chemogenetic interactions from CRISPR screens with drugZ. Genome Med.

[46] Simoneau A (2021). The *trans* cell cycle effects of PARP inhibitors underlie their selectivity toward BRCA1/2-deficient cells. Genes Dev.

[47] Chou TC (2006). Theoretical basis, experimental design, and computerized simulation of synergism and antagonism in drug combination studies. Pharmacol Rev.

[48] Allalou A, Wahlby C (2009). BlobFinder, a tool for fluorescence microscopy image cytometry. Comput Methods Programs Biomed.

