# Supplementary material for: FGFR3-induced Y158 PARP1 phosphorylation promotes PARP inhibitor resistance via BRG1/MRE11-mediated DNA repair in breast cancer models
Source: J Clin Invest. 2025 May 29;135(14):e173757. doi: 10.1172/JCI173757 (PMC12259256; doi:10.1172/JCI173757)
Supplement: Supplemental data [file jci-135-173757-s133.pdf]

Supplemental Information

FGFR3-induced Y158 PARP1 phosphorylation promotes PARP-inhibitor resistance via BRG1/MRE11-mediated DNA repair in breast cancer models

Chen et al.

Table of Contents

1. Supplemental Materials and Methods (Page 3-6)

2. Supplemental Tables (Page 7-8)

Supplemental Table 1. Gene ontology analysis of FGFR3-interacting protein in SUM149 and BR#09 cells.

Supplemental Table 2. Pivot tables for p-Y158 PARP1 and p-FGFR IHC staining in breast cancer patient-derived xenograft mouse models.

Supplemental Table 3. DrugZ analysis of FGFR family members (FGFR1-4).

3. Supplemental Figures (Page 9-27)

Supplemental Figure 1. FGFR3 activation contributes to PARPi resistance in BR cells.

Supplemental Figure 2. FGFR3 mutations are rare in breast cancer.

Supplemental Figure 3. FGFR3 contributes to PARPi resistance in breast cancer cells.

Supplemental Figure 4. Inhibition of FGFR3 sensitizes BR cells to PARPi.

Supplemental Figure 5. Inhibition of FGFR3 enhances DNA damages.

Supplemental Figure 6. FGFR3 interacts with and phosphorylates PARP1 at Y158.

Supplemental Figure 7. PARP1 p-Y158 enhances DNA damage repair.

Supplemental Figure 8. PARP1 p-Y158 does not increase PARP1 enzymatic activity, but increase chromatin-bound BRG1 and MRE11.

Supplemental Figure 9. Combination of talazoparib and PD173074 inhibits tumor growth in breast cancer mouse models.

Supplemental Figure 10. p-Y158 PARP1 and pFGFR in PDX tumor tissues.

4. References (Page 28)

1. Supplementary Materials and Methods

Cell culture and talazoparib-resistant BR cell development

SUM149 cells (BioIVT) were maintained in F-12K medium (American Type Culture Collection [ATCC], 30-2004) supplied with 5% fetal bovine serum (FBS), 10mM HEPES, 1 µg/ml hydrocortisone, 5 µg/ml insulin, and 100 units/ml penicillin with 100 µg/ml streptomycin (P/S). Other cell lines used were purchased from ATCC. MDA-MB-231, BT549, MDA-MB-468, MDA-MB-157, Hs578T and MCF-7 cells were maintained in Dulbecco modified Eagle medium/F-12 medium (Caisson Laboratories) supplemented with 10% FBS and P/S. HCC70 and HCC1937 cells were maintained in RPMI 1640 medium (Corning) supplemented with 10% FBS and P/S. Cell lines were validated by short tandem repeat DNA fingerprinting using the AmpF_STR Identifiler kit according to the manufacturer's instructions (Applied Biosystems), and the profiles were matched to known ATCC fingerprints (ATCC.org) and to the Cell Line Integrated Molecular Authentication database version 0.1.200808 (http://bioinformatics.istge.it/clima/). SUM149 BR cells were selected by treating SUM149 cells with 100nM talazoparib for 5 consecutive days and then with 15-50nM talazoparib until resistant cells grew into clones. Single clones were cultured in 50nM talazoparib-containing complete F-12K medium until stably proliferating. The cells were then maintained without talazoparib.

Reagents and antibodies

Talazoparib (BMN-673), veliparib (ABT-888), PD173074, erdafitinib, rucaparib, and AZD4547 were purchased from Selleck Chemistry. Olaparib was purchased from LC Laboratories. AZD4547 and PD173074 for animal experiments and BRM/BRG1 ATP Inhibitor-1 were purchased from MedChemExpress. All inhibitors were dissolved in dimethyl sulfoxide (DMSO) or dimethylacetamide to make stock solution. Unless otherwise indicated, 100nM talazoparib and 10µM PD173074 were used for treatment of cells. Methyl methanesulfonate (MMS) was purchased from Sigma Aldrich and a final concentration of 0.01% MMS was used for treatment of cells.

The primary antibodies and dilution ratios for Western blot analysis used in the current study were as follows: mouse anti-FGFR3 (#NBP2-52468; 1:1,000) from Novus Biologicals; rabbit anti-Histone H4 (#ab10158; 1:1,000), rabbit anti-FGFR3 (#ab137084; 1:1,000), and rabbit anti-FGFR3 (phosphor Y724) (#ab155960; 1:1,000) from Abcam; rabbit anti-PARP (#9532S; 1:1,000) from Cell Signaling Technology; rabbit anti-actin (#A2066; 1:5,000), mouse anti-tubulin (#T5158; 1:5,000), mouse anti–phospho-histone H2A.X (Ser139; #05-636; 1:1,000), mouse anti-HA (clone 12CA5; 1:1,000), and rabbit anti–phospho-FGFR (Tyr653/Tyr654; #06-1433; 1:1000) from MilliporeSigma; and mouse anti-FGFR3 (#sc-390423, 1:1,000), rabbit anti-lamin B1 (#sc-374015; 1:2,000), and mouse anti-GAPDH (#sc-32233; 1:1,000) from Santa Cruz Biotechnology.

MTT assay

Cells were seeded at a concentration of 1,000 cells/well in a 96-well plate and cultured overnight before treatments with inhibitors. Inhibitor-containing media was refreshed every 3 days for a 6-day treatment schedule. Cells were incubated with 0.5 mg/ml thiazolyl blue tetrazolium bromide (Sigma-Aldrich) for 2 hours, and formazan crystals were dissolved using DMSO. Optical density at 565 nm was measured and survival percentages were calculated by normalizing the optical density value of each treatment group to that of the control group, treated with DMSO only. Half-maximal inhibitory

concentrations of inhibitors were calculated using the standard curve interpolate function in GraphPad Prism 8.

Colony formation assay

SUM149 cells (600 cells/well), BR#09 cells (800 cells/well), and BR#17 cells (1,000 cells/well) were seeded into a 12-well plate 18 hours before treatments. Inhibitor-containing media was refreshed every 2 days. Cells were fixed using 4% paraformaldehyde after 10-14 days of treatment. Colonies were stained with 0.5% crystal violet before plates were imaged, and colony number was quantified using the Celigo imaging cytometer (Nexcelom Bioscience). Cell survival rate was calculated by normalizing the number of colonies in each well to that of the vehicle-treated well on the same culture plate.

Immunoprecipitation and Western blot analysis

For immunoprecipitation, cells were treated and lysed with lysis buffer (20mM Tris [pH 8.0], 137mM NaCl, 1% Nonidet P-40, and 2mM EDTA) before 500 µg of proteins was diluted to 500 µL with lysis buffer and incubated with 2.5 µg primary antibodies overnight. The precipitated protein complex was then washed and subjected to Western blot analysis performed as described previously (1). Signals were detected using the ImageQuant 4000 system (GE Healthcare) and quantified using Image Studio Lite.

Receptor tyrosine kinase antibody array

A Proteome Profiler Human Phospho-RTK Array Kit (R&D Systems, #ARY001B) was used according to the manufacturer's instructions. In brief, cells were treated with DMSO or 100nM talazoparib overnight and then harvested for antibody array analysis. Signal data from the array were captured and analyzed as Western blot images. Signals on each array were normalized to the mean signal value of reference controls.

Proximity ligation assay and immunofluorescence staining

Cells were treated for 1 hour with 0.01% MMS, 0.1µM talazoparib, or 10µM PD173074 as indicated before being fixed with 4% paraformaldehyde. PD173074 was introduced 2-4 hours before it was combined with other chemicals, to ensure that FGFR3 was inhibited while inducing DNA damage. The proximity ligation assay (Duolink In Situ Red, Sigma Aldrich) was performed following the manufacturer's instructions. Mouse anti-PARP1 (Sino Biological, #11040-MM04) and rabbit anti-FGFR3 (Abcam, #ab137084) primary antibodies for the proximity ligation assay were diluted at a ratio of 1:500 and incubated with samples overnight at 4 °C.

Immunofluorescence staining and confocal microscopy were performed as previously described (1). Cells were treated with DMSO (solvent control), talazoparib (125nM for BR#09; 250nM for BR#17), PD173074 (10µM), or the combination of talazoparib and PD173074 for the time indicated. Primary antibodies were diluted in 5% bovine serum albumin at a ratio of 1:500 for both anti–phospho-histone H2A.X (γH2AX) and anti-FGFR3 and were incubated overnight. Secondary antibodies anti-mouse fluorescein isothiocyanate and anti-rabbit TexasRed were diluted at a 1:1,000 ratio in 5% bovine serum albumin. In both immunostaining and the proximity ligation assay, images of the cells were captured and analyzed with an LSM 710 laser confocal microscope and Zeiss Zen software (Carl Zeiss) and foci counting was performed using BlobFinder (2).

Cloning and mutagenesis

FGFR3-expressing plasmid pDONR223_FGFR3 (Addgene plasmid #23933) was a gift of Dr. William Hahn and Dr. David Root (3). FGFR3 was subcloned from pDONR223-FGFR3 into pCDH-CMV-MCS-EF1-Neo (System Biosciences) by amplifying the *FGFR3* open reading frame with polymerase chain reaction. 3xFlag-tag was inserted by oligomer annealing. HA-tagged PARP1 expression plasmid was described in our previous study (4). $PARP1^{Y158D}$-, $PARP1^{Y158F}$- and $PARP1^{Y176F}$-expressing plasmids were generated using site-directed mutagenesis polymerase chain reaction and HA-PARP1 plasmid (4). Cells with transient expression of the $PARP1^{Y158D}$ plasmid, stable expressions of the $PARP1^{Y158F}$ and $PARP1^{Y176F}$ were selected by 500 µg/ml G418 (Thermo Fisher).

RNA interference and stable cell lines

PARP1-targeting short hairpin RNAs (shRNAs; shPARP1-1: TRCN0000007928; shPARP1-2: TRCN0000356550), BRCA1-targeting shRNAs (shBRCA1-1: TRCN0000009823; shBRCA1-3: TRCN0000039833), and FGFR3-targeting shRNAs (TRCN0000000371 and TRCN0000196809) were purchased from Sigma Aldrich. FGFR3-targeting shRNAs were subcloned into EZ-Tet-pLKO-Puro (Addgene plasmid #85966), a gift from Dr. Cindy Miranti (5). Lentivirus particles were generated by transfecting HEK293T cells with pCMV-VSV-G (Addgene plasmid #8454), pCMV-dR8.91, and shRNA plasmids, PARP1-expressing plasmids, or FGFR3-expressing plasmids in a 1:3:6 ratio. Scramble shRNA control plasmid pLKO.1 (Addgene plasmid #1864) was a gift from David Sabatini and pCMV-VSV-G was a gift from Dr. Bob Weinberg. Stable cells were selected and maintained in the selection medium containing 1 µg/ml puromycin (InvivoGen) or 500 µg/ml G418 (Thermo Fisher).

Comet assay

Cells were seeded into a 60-mm cell culture dish at least 18 hours before reaching 60% confluence for treatments. Cells were treated with 0.01% MMS, 100nM talazoparib, and 10µM PD173074 as indicated for 1 hour before MMS removal. For cell release from MMS, culture medium was removed and cells were washed with ice-cold phosphate-buffered saline twice before freshly prepared inhibitor-containing medium was added for DNA repair. The alkaline comet assay was performed as described previously (6). DNA damage was further digested with 2U formamidopyrimidine [fapy]-DNA glycosylase (New England BioLabs, #M0240S) for 1 hour before electrophoresis (22 V, 300 mA, 20 minutes). Comet olive moment was measured using CometScore v1.5 (TriTek).

PARP trapping assay

Chromatin-bound PARP1 was isolated as previously described (7, 8) with some modifications. Cells were treated with or without 10µM PD173074 for at least 4 hours before treatment with 100nM talazoparib and 0.1% MMS. After treatment with MMS, cells were washed with ice-cold phosphate-buffered saline twice before incubation with fresh normal culture medium or PD173074-containing medium for the time indicated. Cells were then trypsin-harvested and lysed with HDG150 buffer before the chromatin fraction was incubated in HDG150 buffer with 5mM $CaCl_2$ and 100 U/ml micrococcal nuclease for 1 hour at 4 °C. Supernatant of chromatin fraction was then subjected to Western blot analysis.

Mouse models

Animal studies were performed following a protocol approved by The University of Texas MD Anderson Cancer Center Institutional Animal Care and Use Committee. Female nude mice were purchased from the Department of Experimental Radiation Oncology at MD Anderson. For BR#09 and BR#17 xenograft mouse models, two million cells were mixed with 50% (v/v) growth factor reduced Matrigel matrix (Corning) and inoculated into the mammary fat pads of 6- to 8-week-old female nude mice. For PDX xenograft mouse models, the tumor chunks of TNBC PDX model (BCX.070) were inoculated into the 4th mammary fat pad of 6- to 8-week-old female nude mice. For BT549 xenograft mouse models, two million cells were mixed with 50% (v/v) growth factor reduced Matrigel matrix and inoculated into the mammary fat pads of 6- to 8-week-old female nude mice. For the 4T1 model, female Balb/c mice were purchased from Jackson Laboratory. A total of 50,000 4T1 cells were mixed with Matrigel matrix and inoculated into the mammary fat pad of 6-week-old female Balb/c mice. Inhibitors were dissolved in vehicle solvent containing 10% dimethylacetamide (Sigma-Aldrich), 5% Kolliphor HS 15 (Sigma-Aldrich), and 85% phosphate-buffered saline (9). Final concentrations of the inhibitors used in the mouse models are as follows: talazoparib (0.25 mg/kg per day), PD173074 (15 mg/kg per day), olaparib (40 mg/kg per day), and AZD4547 (8 mg/kg per day). Treatment with inhibitors started when tumor volumes reached a mean of 120 mm^3. Mice were treated using oral gavage daily for 20 days followed by 3 days with no drugs to prevent severe weight loss. After the first cycle, treatment was continued on a schedule of 6 days on and 1 day off. Mouse weight and tumor volume were measured three times every week. Tumor volume was estimated using the following formula: volume (mm^3) = length (mm) × width (mm) × 0.5 width (mm), where length is the longest axis of the tumor. Mice were euthanized using CO_2 when the tumor volume reached 2,000 mm^3. For the blood chemical test, mouse cardiac blood was collected by veterinarians in the Department of Veterinary Medicine and Surgery at MD Anderson after 16 days of treatment. Concentrations of alanine aminotransferase, aspartate aminotransferase, and blood urea nitrogen in Balb/c mice were referred to North American colonies of Charles River Balb/c mice (https://www.criver.com/sites/default/files/resources/BALBcNudeMouseClinicalPathologyData.pdf).

2. Supplemental Tables

Supplemental Table 1. Gene ontology analysis of FGFR3-interacting protein in SUM149 and BR#09 cells.

Analysis Type:	PANTHER Overrepresentation Test (Released 20200728)
Annotation Version and Release Date:	GO Ontology database DOI: 10.5281/zenodo.4081749 Released 2020-10-09
Reference List:	Homo sapiens (all genes in database)
Test Type:	FISHER
Correction:	FDR

GO molecular function complete	REFLIST (20595)	BR#09 only (231)	Expected	over/under	Fold Enrichment	raw P-value	FDR
structural constituent of ribosome (GO:0003735)	167	36	1.87	+	19.22	6.04E-33	5.77E-30
nucleosomal DNA binding (GO:0031492)	39	10	0.44	+	22.86	1.31E-10	4.46E-08
miRNA binding (GO:0035198)	30	7	0.34	+	20.8	1.46E-07	2.69E-05
telomerase RNA binding (GO:0070034)	22	4	0.25	+	16.21	1.82E-04	1.77E-02
RNA cap binding (GO:0000339)	19	5	0.21	+	23.46	5.75E-06	7.85E-04
ribosomal small subunit binding (GO:0043024)	17	4	0.19	+	20.98	7.61E-05	8.87E-03
RNA 7-methylguanosine cap binding (GO:0000340)	12	4	0.13	+	29.72	2.42E-05	3.12E-03
protein-arginine N-methyltransferase activity (GO:0016274)	11	3	0.12	+	24.32	4.48E-04	3.75E-02
arginine N-methyltransferase activity (GO:0016273)	11	3	0.12	+	24.32	4.48E-04	3.69E-02
RNA strand annealing activity (GO:0033592)	5	3	0.06	+	53.49	7.24E-05	8.65E-03

GO molecular function complete	REFLIST (20595)	SUM149 (144)	Expected	over/under	Fold Enrichment	raw P-value	FDR
pre-mRNA binding (GO:0036002)	39	9	0.27	+	33	3.88E-11	1.69E-08
poly-pyrimidine tract binding (GO:0008187)	28	8	0.2	+	40.86	1.14E-10	4.19E-08
mRNA 3'-UTR AU-rich region binding (GO:0035925)	27	7	0.19	+	37.08	3.09E-09	9.84E-07
AU-rich element binding (GO:0017091)	28	7	0.2	+	35.76	3.84E-09	1.15E-06
poly-purine tract binding (GO:0070717)	29	7	0.2	+	34.52	4.74E-09	1.33E-06
poly(A) binding (GO:0008143)	23	6	0.16	+	37.31	4.20E-08	1.00E-05
poly(U) RNA binding (GO:0008266)	25	6	0.17	+	34.33	6.44E-08	1.34E-05
pre-mRNA intronic binding (GO:0097157)	12	4	0.08	+	47.67	3.80E-06	6.49E-04
U1 snRNA binding (GO:0030619)	8	3	0.06	+	53.63	5.19E-05	6.89E-03
U5 snRNA binding (GO:0030623)	2	2	0.01	+	> 100	2.85E-04	3.24E-02

FGFR3-interacting proteins were analyzed from immunoprecipitated FGFR3 followed by mass spectrum analysis. Gene ontology analysis of the FGFR3-interacting protein lists using the PANTHER Overrepresentation Test was performed using the PANTHER Classification System (http://www.pantherdb.org/). The lists were sorted by the number of proteins in the GO function group detected in the samples.

Supplemental Table 2. Pivot tables for p-Y158 PARP1 and p-FGFR IHC staining in breast cancer patient-derived xenograft mouse models.

ID	H-score	p-Y158 PARP1	Response	ID	H-score	p-FGFR	Response
L16-PDX01	10	Low	Sensitive	L16-PDX01	17	Low	Sensitive
L18-PDX01	6	Low	Sensitive	L18-PDX01	10	Low	Sensitive
L18-PDX02	80	High	Resistance	L18-PDX02	12	Low	Resistance
L27-PDX01	10	Low	Sensitive	L27-PDX01	30	High	Sensitive
L27-PDX02	45	Low	Resistance	L27-PDX02	150	High	Resistance
L34-PDX01	20	Low	Sensitive	L34-PDX01	90	High	Sensitive
BCX.006	279	High	Resistance	BCX.006	7	Low	Resistance
BCX.010	120	High	Resistance	BCX.010	18	Low	Resistance
BCX.011	5	Low	Resistance	BCX.011	1	Low	Resistance
BCX.017	163	High	Resistance	BCX.017	21	Low	Resistance
BCX.022	120	High	Sensitive	BCX.022	19	Low	Sensitive
BCX.024	50	Low	Sensitive	BCX.024	29	High	Sensitive
BCX.051	160	High	Resistance	BCX.051	27	High	Resistance
BCX.060	75	Low	Sensitive	BCX.060	8	Low	Sensitive
BCX.070	30	Low	Resistance	BCX.070	4	Low	Resistance
BCX.080	125	High	Sensitive	BCX.080	22	High	Sensitive
BCX.087	70	Low	Resistance	BCX.087	18	Low	Resistance
BCX.092	150	High	Resistance	BCX.092	23	High	Resistance
BCX.094	150	High	Resistance	BCX.094	47	High	Resistance
BCX.095	60	Low	Sensitive	BCX.095	46	High	Sensitive
BCX.096	90	High	Resistance	BCX.096	28	High	Resistance
BCX.102	90	High	Resistance	BCX.102	33	High	Resistance

The H-score of the PDX model IHC staining results were calculated by multiplying the intensity (0-3) of the staining with the percentage of cells carrying positive signals (0-100). The H-score above the median is defined as high, and below the median is defined as low. The response of these PDX models to talazoparib treatment were shown in the previous study (9).

Supplemental Table 3. DrugZ analysis of FGFR family members (FGFR1-4).

Receptor	FGFR1	FGFR2	FGFR3	FGFR4
normZ	0.83	0.3	-0.98	-0.13
p	0.796	0.617	0.163	0.447

FGFR1-4 receptors were knocked out from SUM149-derived talazoparib-resistant BR#19 cells using a pooled CRISPR library (Horizon Discovery Ltd.) provided by the Functional Genomics Core Facility at The University of Texas MD Anderson Cancer Center. The DrugZ analysis (10) was performed to compare control cells with cells that survived after treatment with talazoparib. A negative normZ score indicates synergistic lethality of the gene with talazoparib.

3. Supplemental Figures

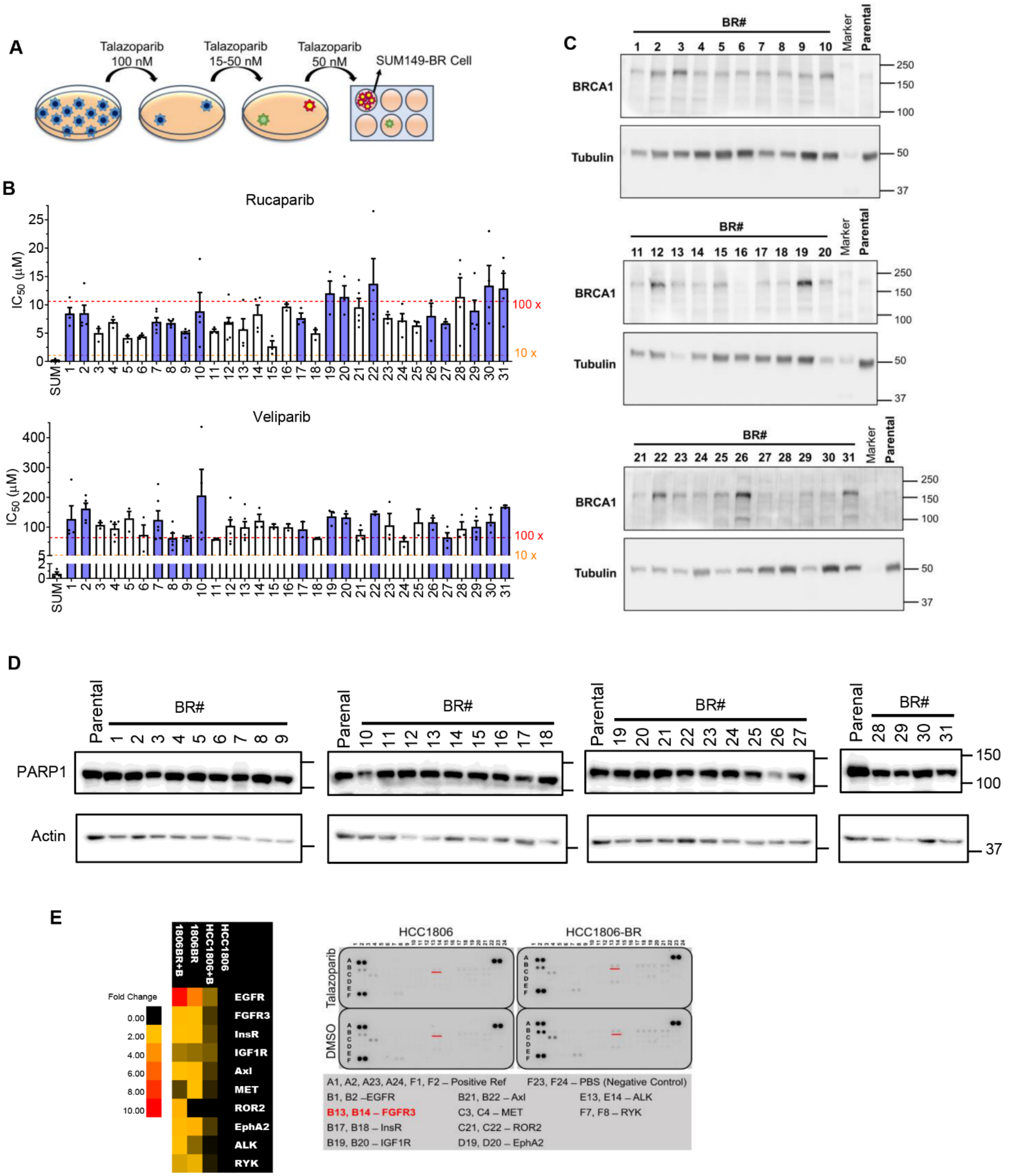

Supplemental Figure 1. FGFR3 activation contributes to PARP inhibitor (PARPi) resistance in BR cells. (A) Illustration of BR cell development. **(B)** Half-maximal inhibitor concentration (IC_{50}) of

rucaparib and veliparib in parental SUM149 cells (SUM) and 31 BR cells, measured by MTT assay. Cells were treated with various concentrations of PARPi for 6 days before cell survival was analyzed by MTT assay. IC_{50} was calculated using GraphPad Prism 8.0. Fold-change (x) of IC_{50} was compared with that of SUM149 parental cells. Histograms show mean ± S.E.M. ($n \geq 3$). **(C)** Full-length BRCA1 protein expression in SUM149 parental cells and the BR cells. Western blot analysis was performed with antibody against C-terminal BRCA1 (Cell Signaling Technology, #14823). **(D)** PARP1 and actin expression in the BR clones were determined by western blot analysis. **(E)** Antibody arrays of RTK activation in HCC1806 and HCC1806-BR cells. Cells indicated were treated with DMSO or 100nM talazoparib overnight and harvested for RTK antibody array analysis. Signal intensities from RTK antibody arrays were compared with those of parental cells, and shown as the heatmaps in the left.

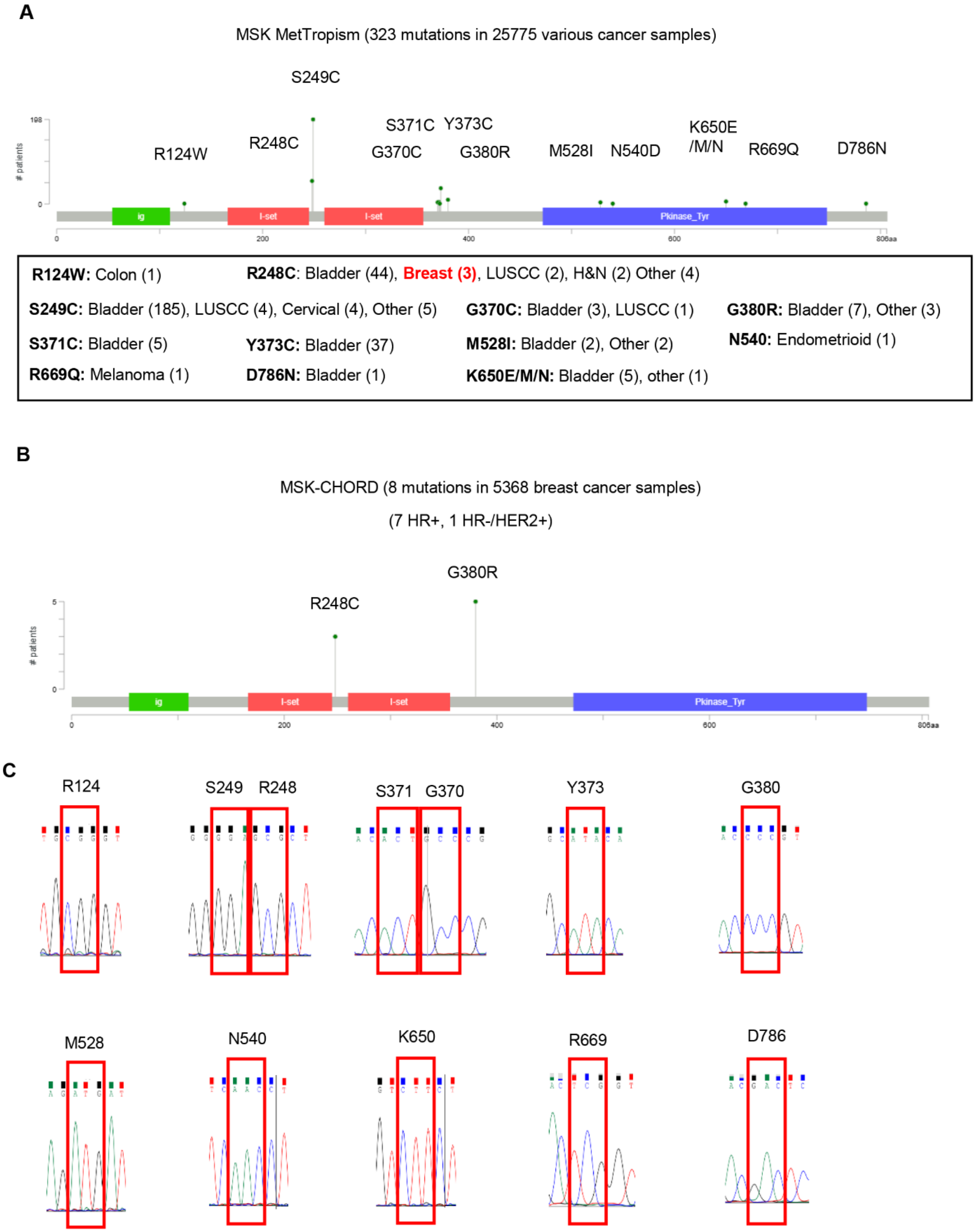

Supplemental Figure 2. *FGFR3* mutations are rare in breast cancer. (A, B) *FGFR3* mutations associated with cancer were analyzed in cBioportal. **(A)** 323 patient samples have *FGFR3* mutations associated with cancer in MSK MetTropism (25,775 samples from various cancer patients) as shown

below. **(B)** 8 patient samples have FGFR3 mutations associated with cancer in breast cancer patient samples of MSK-CHORD (5368 breast cancer samples). Among the 8 samples, seven are in hormone receptor (HR) positive breast cancer and one is in HR negative/HER2 positive breast cancer. **(C)** Analysis of the sequence of SUM149 BR cells. Genomic DNA was isolated from 5 of SUM149 BR cells and subjected to PCR and sanger sequencing. The sequences of S249~G380, K650, and R669 were determined from reverse direction.

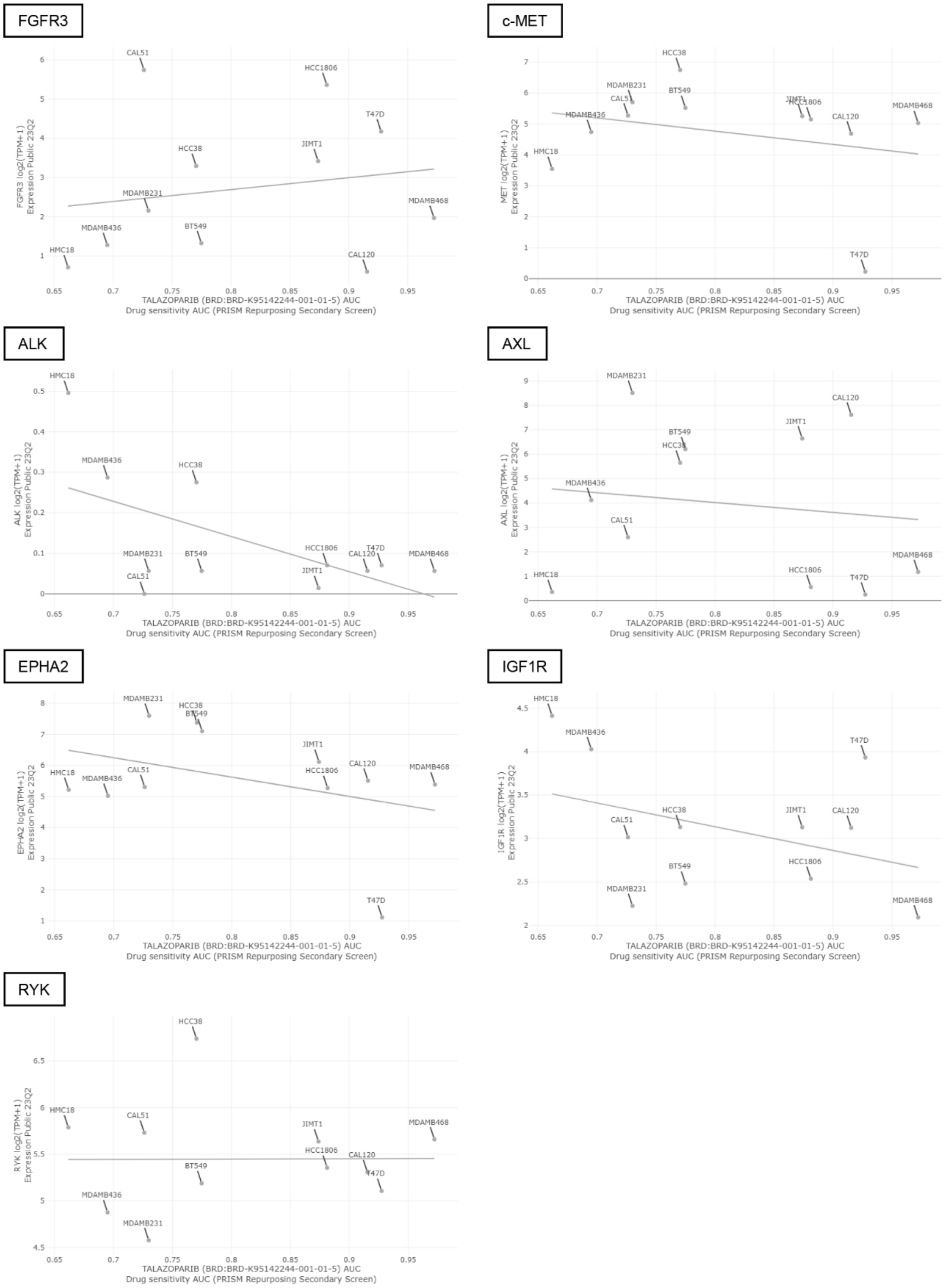

Supplemental Figure 3. FGFR3 contributes to PARPi resistance in breast cancer cells. Expression level of our candidate RTKs and the talazoparib response of breast cancer cell lines were queried in DepMap database, results are plotted and the linear correlation line is shown on each panel.

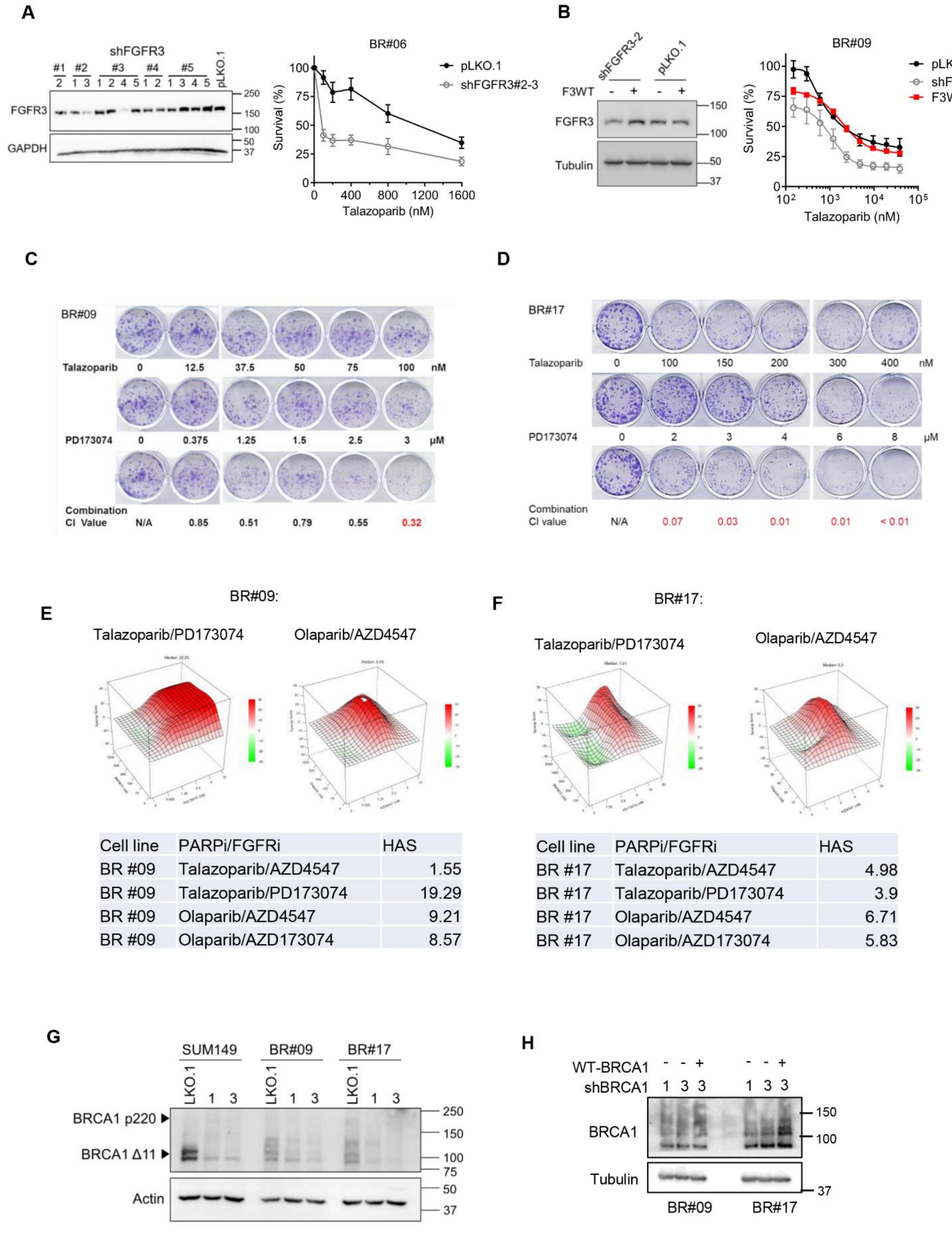

Cell line	PARPi/FGFRi	HAS
BR #09	Talazoparib/AZD4547	1.55
BR #09	Talazoparib/PD173074	19.29
BR #09	Olaparib/AZD4547	9.21
BR #09	Olaparib/AZD173074	8.57

Cell line	PARPi/FGFRi	HAS
BR #17	Talazoparib/AZD4547	4.98
BR #17	Talazoparib/PD173074	3.9
BR #17	Olaparib/AZD4547	6.71
BR #17	Olaparib/AZD173074	5.83

Supplemental Figure 4. Inhibition of FGFR3 sensitizes BR cells to PARPi. (A) Five short-hairpin RNAs (shRNAs) targeting FGFR3 (shFGFR3) were used to knock down endogenous FGFR3 expression in BR#06 cells. Cells transfected with non-target scramble shRNA carrying plasmid (pLKO.1) were introduced as a control group. Single clones were picked from the shRNA-transfected cells, and FGFR3 expression levels were detected by Western blot analysis. Clone 3 from shFGFR3#2 cells was chosen for comparison with control cells to analyze cell survival in response to talazoparib, by MTT assay. The viability at 0 nM of talazoparib was set to 100% for both pLKO and shFGFR3 cells, and the viability values presented are shown relative to this baseline. Data are shown as mean ± S.E.M. ($n \geq 3$). **(B)** FGFR3 expression was knocked down by shFGFR3#2 in BR#09 cells and further rescued by exogenous expression of wild-type FGFR3 (F3WT); expression of FGFR3 was examined by Western blot analysis. The survival rate of the cells indicated in response to talazoparib was analyzed by MTT assay after 6 days of treatment with talazoparib. The viability at 0 nM of talazoparib was set to 100% for both pLKO and shFGFR3 cells, and the viability values presented are shown relative to this baseline. Data are shown as mean ± S.E.M. ($n \geq 3$). **(C, D)** BR#09 and BR#17 cells were treated with talazoparib and PD173074, either alone or in combination, at the concentrations indicated for 10-12 days, and then cells were fixed for the colony formation assay. Representative images of colony formation of BR#09 and BR#17 are shown in C and D, respectively. The quantification of the three independent experiments are shown in Figure 2A. **(E, F)** Synergy between PARPi and FGFRi in BR#09 (E) and BR#17 (F) cells was interrogated with highest single agent model. **(G)** Expression of full-length BRCA1 (BRCA1 p220) and BRCA1 Δ11 in SUM149, BR#09, and BR#17 cells transfected with non-targeting scramble shRNA (LKO.1), BRCA1 shRNA (shBRCA1-1 and shBRCA1-3) was determined by western blot. **(H)** Re-expression of wild type BRCA1 (WT BRCA1) in BR#09 and BR#17 shBRCA1-3 cells was determined by western blot. These cells were used in Figure 2E to determine the CI index to talazoparib plus PD173074.

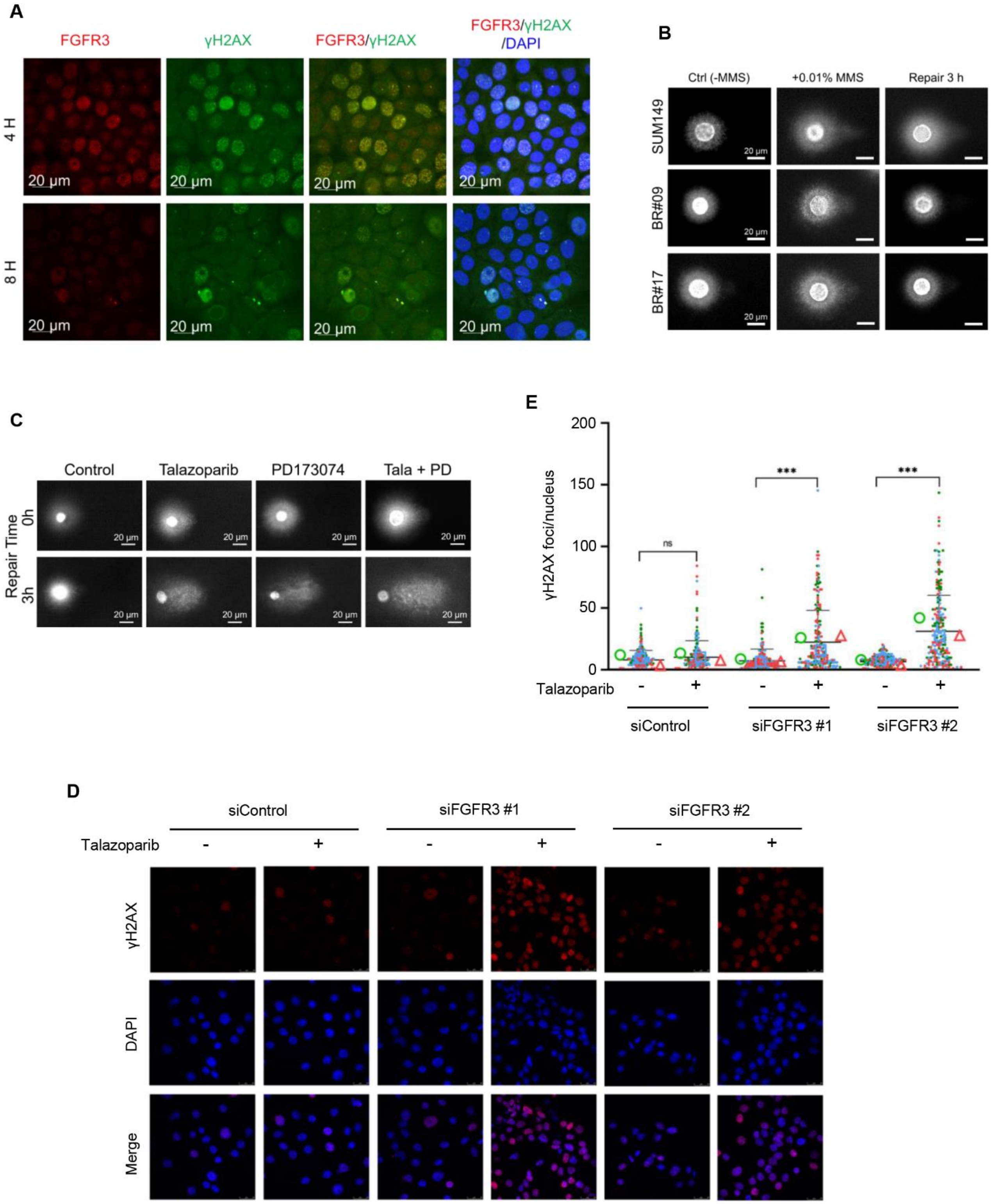

Supplemental Figure 5. Inhibition of FGFR3 enhances DNA damages. (A) BR#09 cells were treated with 200 nM Talazoparib for 4 h and 8 h and subjected to immunofluorescence staining with antibodies against FGFR3 (TexasRed, red) and γH2AX (FITC, green). DNA was counterstained with DAPI (blue).

The images were captured using confocal microscopy. **(B)** Representative images of the comet assay of Figure 3F. **(C)** Representative images of the comet assay of Figure 3G. **(D, E)** FGFR3 is knocked down with two distinct siRNA in BR#17 cells and treated with talazoparib as described in Figure 4D. Then, the cells were subjected to immunofluorescence staining with antibodies against γH2AX (Red). DNA was counterstained with DAPI (blue). Representative images of the cells (D). Dot plots represent mean ± SD from three independent experiments; scatter plots show all cells counted (E). One-way ANOVA Tukey test: ***$p<0.001$; ns, not significant.

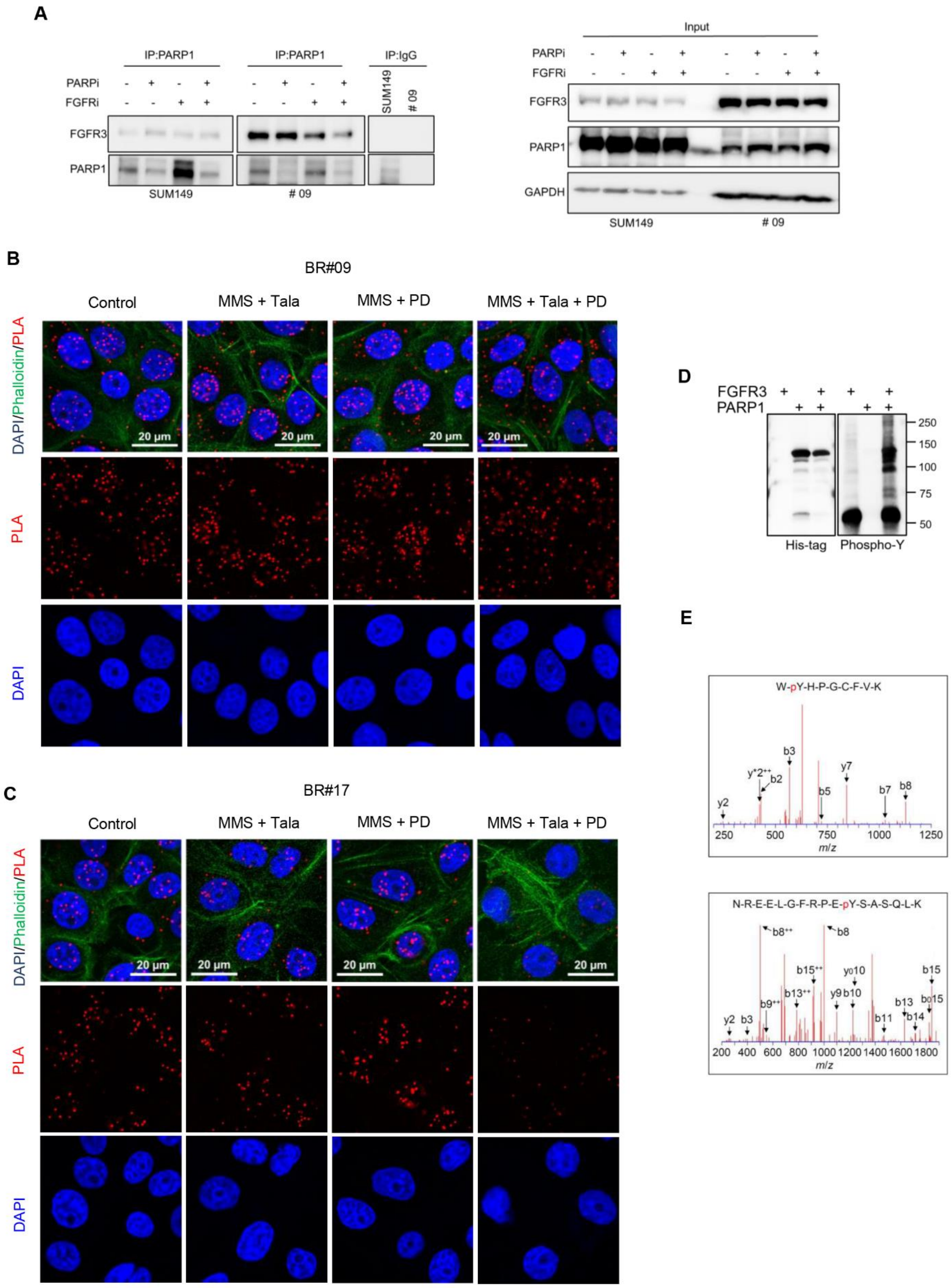

A
IP:PARP1
IP:PARP1
IP:IgG
PARPi
FGFRi
SUM149
09
FGFR3
PARP1
SUM149
09
Input
PARPi
FGFRi
FGFR3
PARP1
GAPDH
SUM149
09
B
BR#09
Control
MMS + Tala
MMS + PD
MMS + Tala + PD
DAPI/Phalloidin/PLA
PLA
DAPI
20 µm
C
BR#17
Control
MMS + Tala
MMS + PD
MMS + Tala + PD
DAPI/Phalloidin/PLA
PLA
DAPI
20 µm
D
FGFR3
PARP1
250
150
100
75
50
His-tag
Phospho-Y
E
W-pY-H-P-G-C-F-V-K
m/z
N-R-E-E-L-G-F-R-P-E-pY-S-A-S-Q-L-K
m/z

Supplemental Figure 6. FGFR3 interacts with and phosphorylates PARP1 at Y158. (A) SUM149-BR#09 cells were treated with 100nM talazoparib (PARPi) and 10µM PD173074 (FGFRi) as indicated before being harvested for PARP1 immunoprecipitation (IP). For each sample, 500 µg total protein lysate was used for immunoprecipitation and 40 µg total protein was used for detecting target proteins in the cell lysate (input). The immunoprecipitated complex was then subjected to Western blot analysis to detect the presence of FGFR3. **(B, C**) The images with PLA signals only, DAPI signals only, and merges of PLA, DAPI and Phalloidin in BR#09 (B) and BR#17 (C) cells treated with PARPi and FGFRi in the presence of MMS. The quantification of the nuclear PLA signals is shown in Figure 4A. **(D)** PARP1 is phosphorylated by FGFR3 *in vitro*. His-tagged PARP1 recombinant protein was incubated either with or without activated FGFR3 kinase domain as indicated before phosphorylated tyrosine residues were detected by Western blot analysis with antibodies against phospho-tyrosine (a mixture of clones 4G10, PY20, and PY100). **(E)** The mass spectrometry analysis identifies phosphorylation at tyrosine residues Y158 and Y176 of PARP1 following *in vitro* kinase assay with FGFR3. MS/MS spectra demonstrating the phosphorylation site assignment at Y158 and Y176.

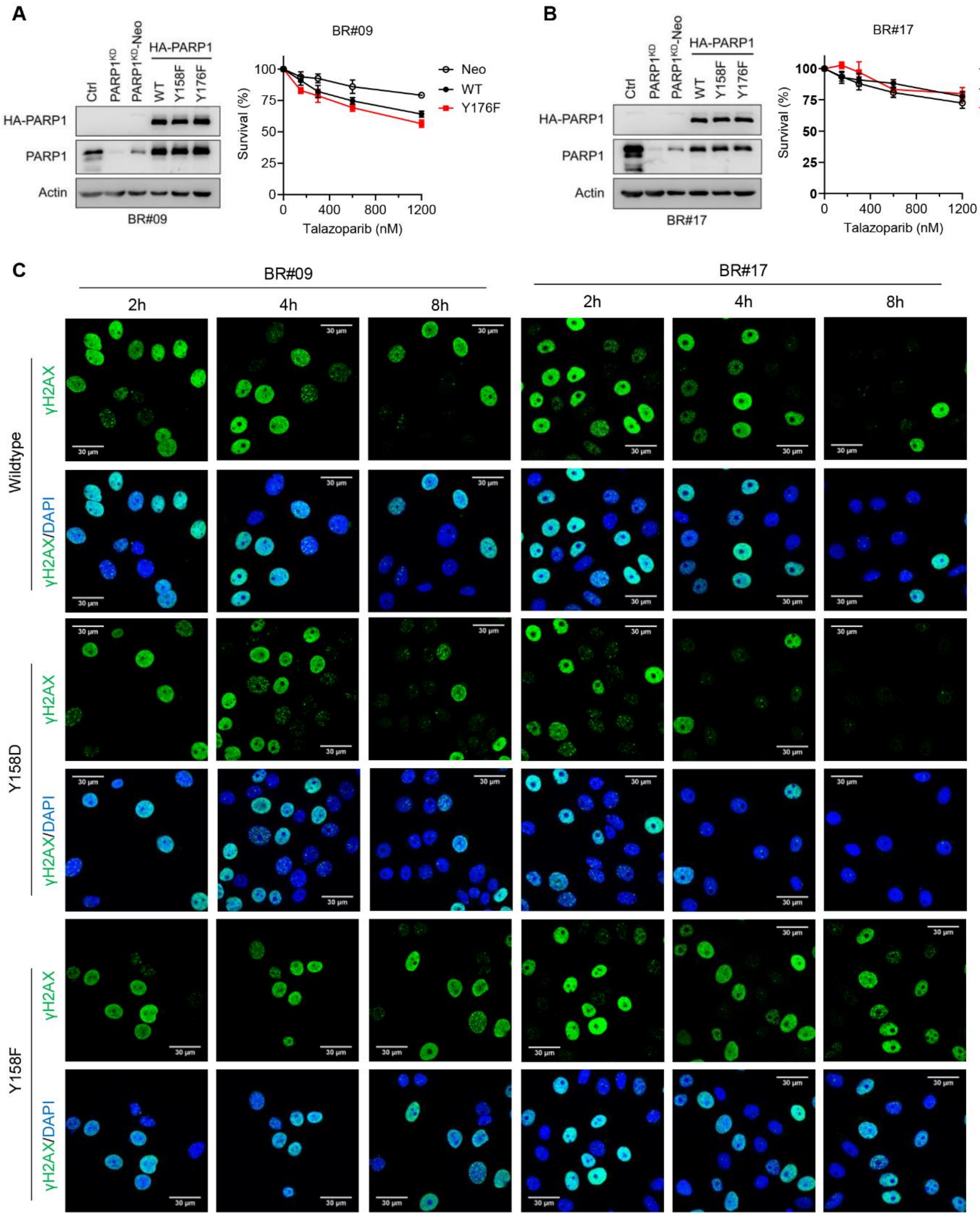

Supplemental Figure 7. PARP1 p-Y158 enhances DNA damage repair. (A, B) PARP1 knockdown (PARP1^{KD}) BR#09 (A) and BR#17 (B) cells were exogenously expressed with hemagglutinin (HA)-tagged vector control (Neo), PARP1^{WT} (WT), PARP1^{Y158F} and PARP1^{Y176F} as indicated. PARP1 expression was then examined by Western blot analysis (The left panels). Cells were treated with various concentrations of talazoparib for 6 days before the cell survival rate was measured by MTT

assay. Means ± standard deviation of three independent experiments are shown in the right graphs (BR#09 (A) and BR#17 (B) cells). **(C)** BR#09 and BR#17 cells expressing $PARP1^{WT}$ (Wildtype), $PARP1^{Y158D}$ (Y158D), or $PARP1^{Y158F}$ (Y158F) were treated with 0.01% MMS and 200nM talazoparib for 30 minutes. Cells were then released from MMS and incubated with 100nM talazoparib for the time indicated (time after MMS removal). Immunofluorescence staining was used to detect γH2AX foci (green) and DNA was stained with DAPI (blue). Scale bar, 30 µm. The quantification is in Figure 4D.

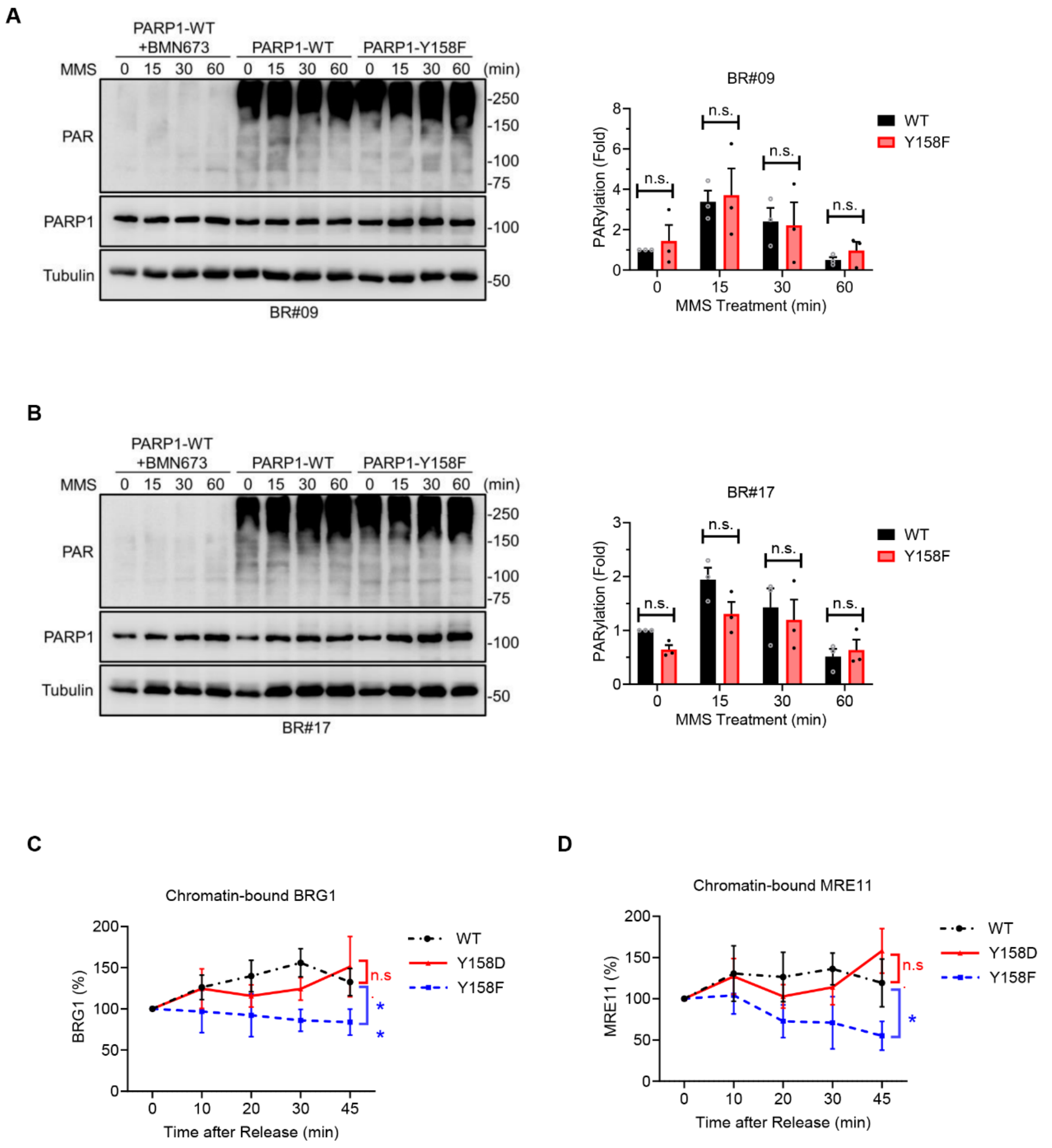

Supplemental Figure 8. PARP1 p-Y158 does not increase PARP1 enzymatic activity, but increase chromatin-bound BRG1 and MRE11. (A, B). PARP1^{Y158F} does not affect MMS-induced PARylation in BR#09 (A) and BR#17 (B) cells. BR#09 and BR#17 cells expressing either PARP1^{WT} (PARP1-WT) or PARP1^{Y158F} (PARP1-Y158F) were treated with 0.01% MMS for 1 hour. PARP1 expression, PARylation (PAR), and tubulin were detected by Western blot analysis (Left panels). PAR signal intensities were normalized to tubulin signal intensity before being compared with wild-type PARP1–expressing cells treated with MMS (1-fold). Histograms show the mean ± standard deviation of three independent repeats. n.s., not significant (Right graphs). **(C)** BR#17 cells expressing PARP1^{WT}, PARP1^{Y158D}, or PARP1^{Y158F} were treated as mentioned in Fig. 5A and harvested at different time points as indicated. Then, chromatin fractions were isolated and subjected to western blot analysis. Chromatin-bound BRG1 and MRE11 signal intensities were normalized to histone H3 and compared with that of

the cells at the beginning of DNA repair (0 minutes after releasing from talazoparib and MMS). Means ± standard deviation from three individual repeats are shown in the histograms. One-way ANOVA Tukey test was used for statistical comparisons. *$p < 0.05$; **$p < 0.01$; n.s., not significant.

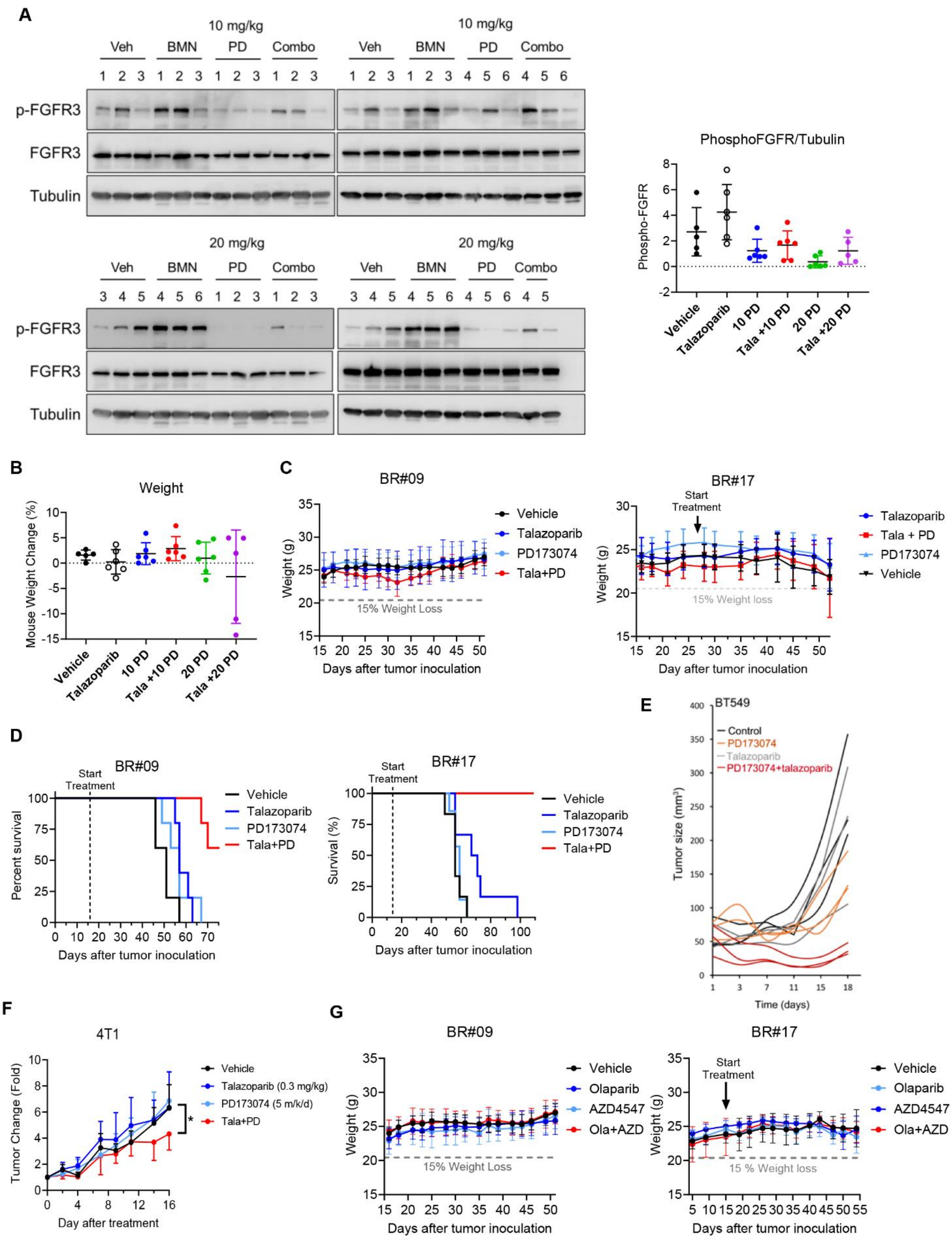
A
10 mg/kg
Veh
BMN
PD
Combo
p-FGFR3
FGFR3
Tubulin
20 mg/kg
PhosphoFGFR/Tubulin
Phospho-FGFR
Vehicle
Talazoparib
10 PD
Tala +10 PD
20 PD
Tala +20 PD
B
Weight
Mouse Weight Change (%)
C
BR#09
BR#17
Vehicle
Talazoparib
PD173074
Tala+PD
Tala + PD
Start Treatment
Weight (g)
15% Weight Loss
15% Weight loss
Days after tumor inoculation
D
Percent survival
Survival (%)
E
BT549
Control
PD173074
Talazoparib
PD173074+talazoparib
Tumor size (mm3)
Time (days)
F
4T1
Tumor Change (Fold)
Talazoparib (0.3 mg/kg)
PD173074 (5 m/k/d)
Day after treatment
G
Olaparib
AZD4547
Ola+AZD
15 % Weight loss

Supplemental Figure 9. Combination of talazoparib and PD173074 inhibits tumor growth in breast cancer mouse models. (A) PD173074 (PD) dose titration in xenograft mouse models. BR#09 cell–derived tumors in xenograft mice (5-6 mice per group) were treated with PD173074 at 10 mg/kg or 20 mg/kg daily for 3 days before tumors were harvested for Western blot analysis. Signal intensities of phosphorylated FGFR (p-FGFR) were normalized to that of tubulin and compared with the mean intensity in a vehicle-treated group, shown in the quantitation panel (mean ± standard deviation). **(B)** Mouse body weight change (mean ± standard deviation) after treatment was normalized to that of the mouse before treatment. **(C)** Animal weights (mean ± standard deviation) in the BR#09 and BR#17 xenograft mouse models treated with 0.25 mg/kg talazoparib per day and 15 mg/kg PD173074 per day either alone or in combination. BR#09 (left) and BR#17 (right). **(D)** Survival curves for the BR#09 and BR#17 xenograft mouse models shown in (C). BR#09 (left) and BR#17 (right). **(E)** Mice bearing tumors of BT549 cells (n = 3) were treated with talazoparib and/or PD173074. Tumor volumes were measured and statistical analysis (One-way ANOVA Tukey test) was performed (talazoparib vs combination: p=0.003, PD173074 or control vs combination: P<0.0001, Other: not significant). **(F)** 4T1 cells (0.5 x 10^6) were injected into the mammary fat pad of Balb/c mice. The indicated treatment was started at the time that the tumors reached an average size of 100 mm^3. Because the tumor sizes varied widely within groups, all tumors were normalized to their own size on the day treatment started. Mice were then euthanized and subjected to blood chemical tests after 17 days of treatment. Mean ± standard deviation of 9 mice is shown (*$p < 0.05$). **(G)** Animal weights in the BR#09 and BR#17 xenograft mouse models treated with 40 mg/kg olaparib per day and 8 mg/kg AZD4547 per day either alone or in combination. Mean ± standard deviation of 5 mice is shown. BR#09 (left) and BR#17 (right).

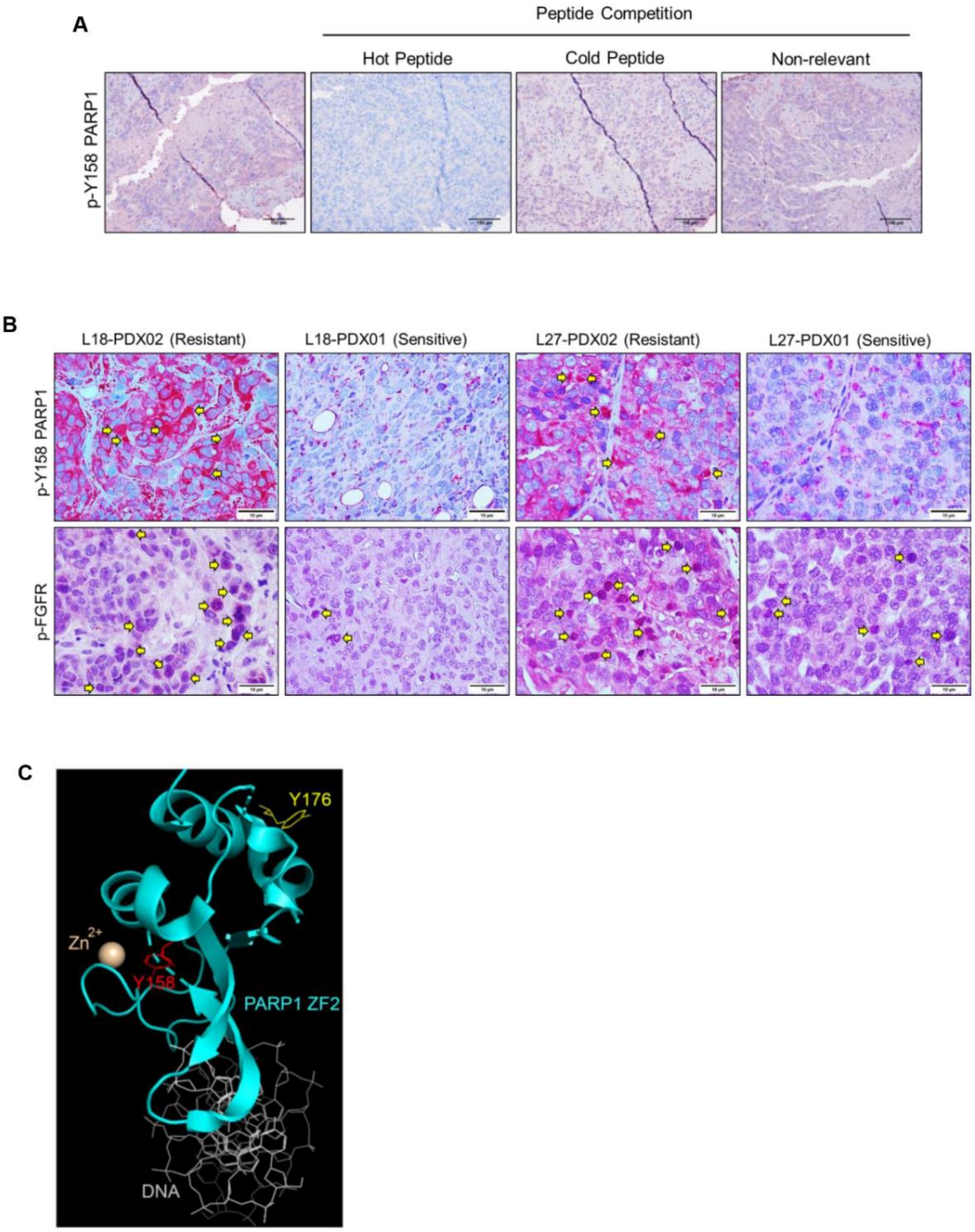

Supplemental Figure 10. p-Y158 PARP1 and pFGFR in PDX tumor tissues. (A) Immunohistochemistry staining with aminoethyl carbazole chromogen for monoclonal antibody against p-Y158 PARP1 was validated in TNBC patient tumors by blocking the tumor tissues with the peptides indicated. Scale bar, 10 µm. Yellow arrowheads indicate cells with positive p-Y158 PARP1 signal in the nucleus. **(B)** p-FGFR and p-Y158 PARP1 immunohistochemistry staining in PDX tumors derived from the naïve (PDX01) and post-talazoparib treated (PDX02) tumor tissues of two TNBC patients (L18 and L27) in a previous talazoparib clinical trial (11). Scale bar, 10 µm. Yellow arrowheads indicate cells with positive p-Y158 PARP1 signal in the nucleus. **(C)** Position of Y158 and Y176 in the PARP1 zinc finger 2 domain. The positions of Y158 and Y176 have been emphasized in previously published crystal

structures of the DNA-bound PARP1 ZF2 domain (PDB: 3ODC) (12). Y158 (red) and Y176 (yellow) are shown here as lines, PARP1 ZF2 (blue) is shown as a cartoon backbone, zinc ion (orange) is shown as a sphere, and DNA (white) is shown as a line.

4. References

1. Chen MK, Du Y, Sun L, Hsu JL, Wang YH, Gao Y, et al. H2O2 induces nuclear transport of the receptor tyrosine kinase c-MET in breast cancer cells via a membrane-bound retrograde trafficking mechanism. *J Biol Chem.* 2019;294(21):8516-28.
2. Allalou A, and Wahlby C. BlobFinder, a tool for fluorescence microscopy image cytometry. *Comput Methods Programs Biomed.* 2009;94(1):58-65.
3. Johannessen CM, Boehm JS, Kim SY, Thomas SR, Wardwell L, Johnson LA, et al. COT drives resistance to RAF inhibition through MAP kinase pathway reactivation. *Nature.* 2010;468(7326):968-72.
4. Du Y, Yamaguchi H, Wei Y, Hsu JL, Wang HL, Hsu YH, et al. Blocking c-Met-mediated PARP1 phosphorylation enhances anti-tumor effects of PARP inhibitors. *Nat Med.* 2016;22(2):194-201.
5. Frank SB, Schulz VV, and Miranti CK. A streamlined method for the design and cloning of shRNAs into an optimized Dox-inducible lentiviral vector. *BMC Biotechnol.* 2017;17(1):24.
6. Chen MK, Tsai YC, Li PY, Liou CC, Taniga ES, Chang DW, et al. Delay of gap filling during nucleotide excision repair by base excision repair: the concept of competition exemplified by the effect of propolis. *Toxicol Sci.* 2011;122(2):339-48.
7. Okada M, Cheeseman IM, Hori T, Okawa K, McLeod IX, Yates JR, 3rd, et al. The CENP-H-I complex is required for the efficient incorporation of newly synthesized CENP-A into centromeres. *Nat Cell Biol.* 2006;8(5):446-57.
8. Murai J, Huang SY, Das BB, Renaud A, Zhang Y, Doroshow JH, et al. Trapping of PARP1 and PARP2 by Clinical PARP Inhibitors. *Cancer Res.* 2012;72(21):5588-99.
9. Evans KW, Yuca E, Akcakanat A, Scott SM, Arango NP, Zheng X, et al. A Population of Heterogeneous Breast Cancer Patient-Derived Xenografts Demonstrate Broad Activity of PARP Inhibitor in BRCA1/2 Wild-Type Tumors. *Clin Cancer Res.* 2017;23(21):6468-77.
10. Colic M, Wang G, Zimmermann M, Mascall K, McLaughlin M, Bertolet L, et al. Identifying chemogenetic interactions from CRISPR screens with drugZ. *Genome Med.* 2019;11(1):52.
11. Litton JK, Scoggins ME, Hess KR, Adrada BE, Murthy RK, Damodaran S, et al. Neoadjuvant Talazoparib for Patients With Operable Breast Cancer With a Germline BRCA Pathogenic Variant. *J Clin Oncol.* 2020;38(5):388-94.
12. Langelier MF, Planck JL, Roy S, and Pascal JM. Crystal structures of poly(ADP-ribose) polymerase-1 (PARP-1) zinc fingers bound to DNA: structural and functional insights into DNA-dependent PARP-1 activity. *J Biol Chem.* 2011;286(12):10690-701.
