# Supplementary figures and images for: FGFR3-induced Y158 PARP1 phosphorylation promotes PARP inhibitor resistance via BRG1/MRE11-mediated DNA repair in breast cancer models
Source: J Clin Invest. 2025 May 29;135(14):e173757. doi: 10.1172/JCI173757 (PMC12259256; doi:10.1172/JCI173757)

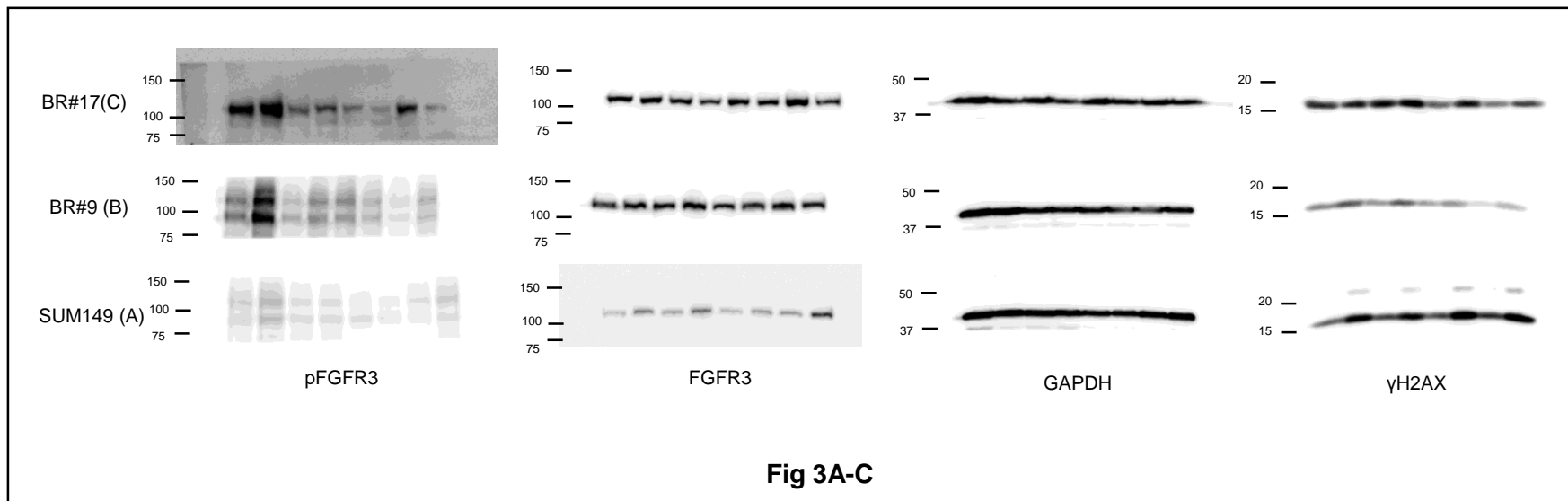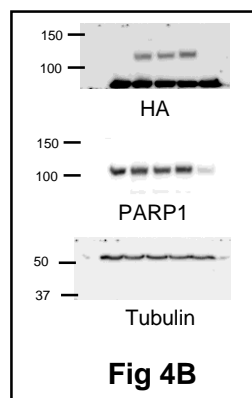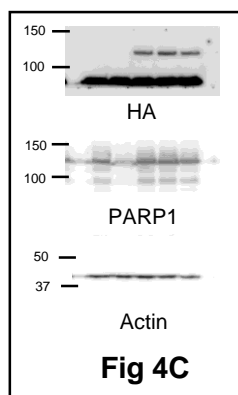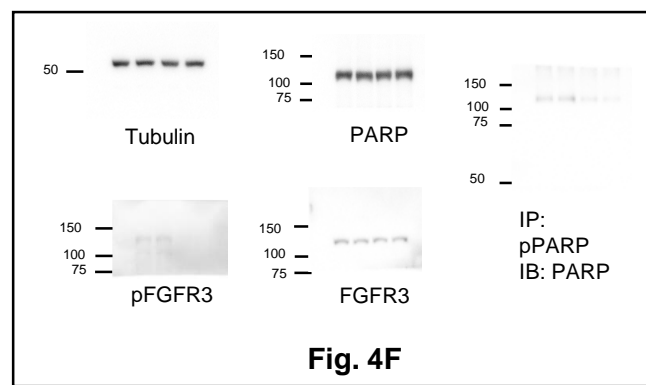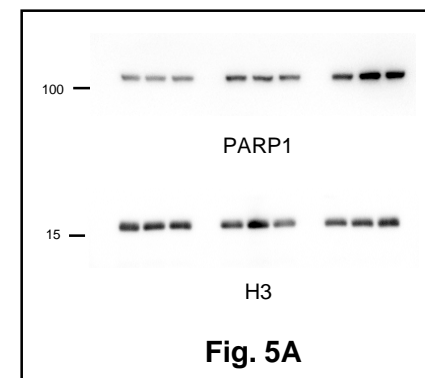

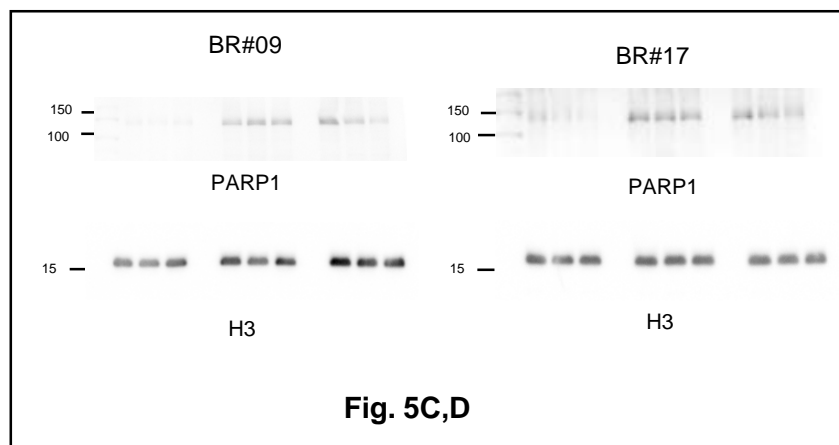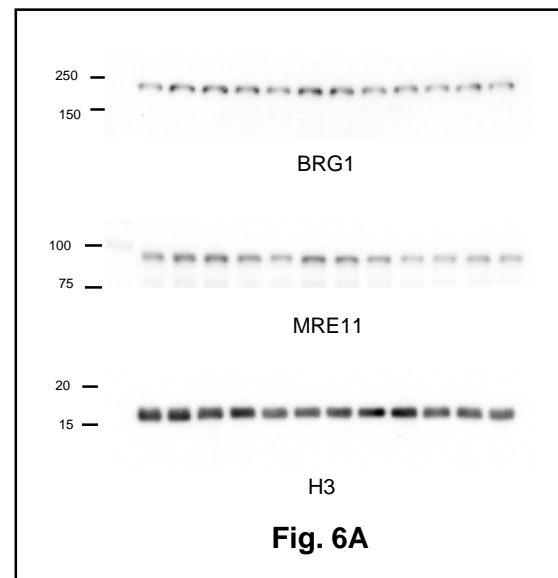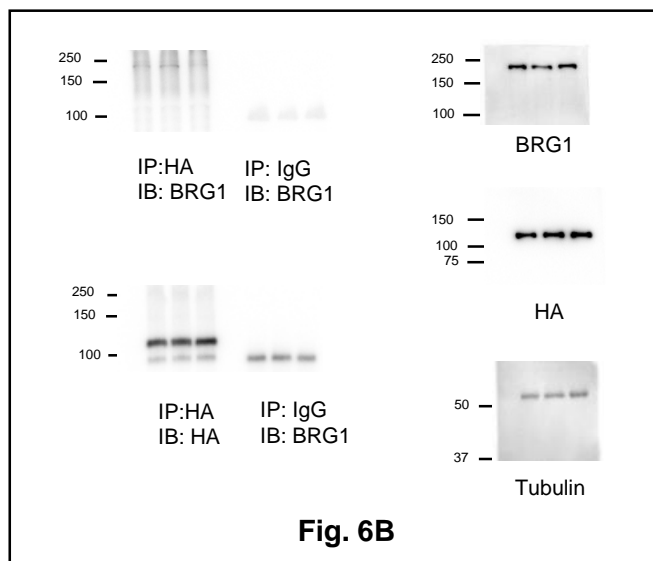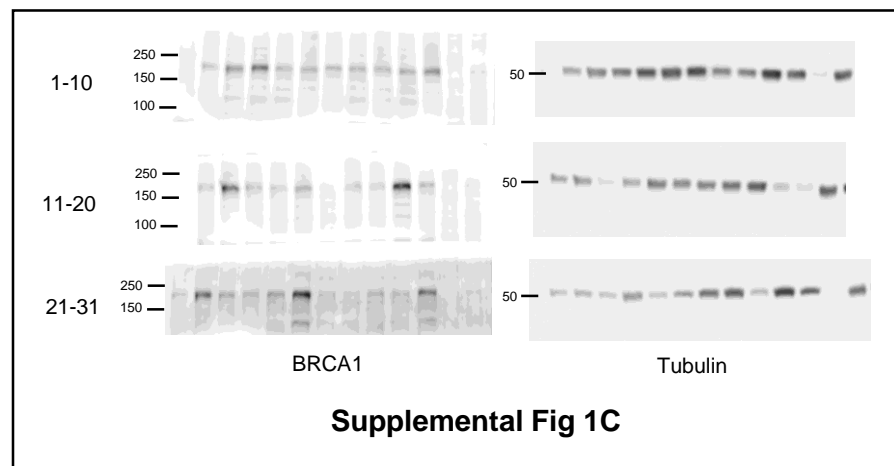

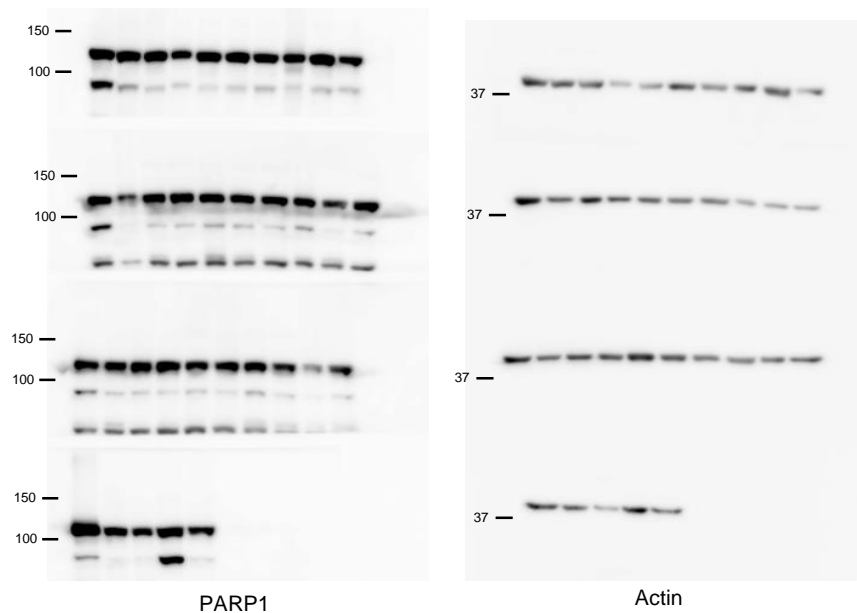

**Supplemental Fig 1D**

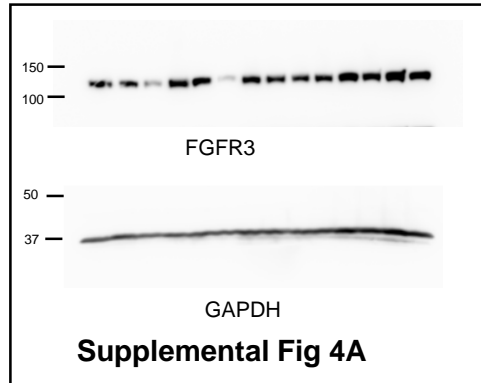

**Supplemental Fig 4A**

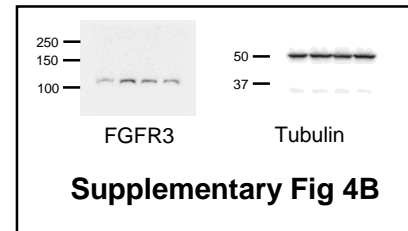

**Supplementary Fig 4B**

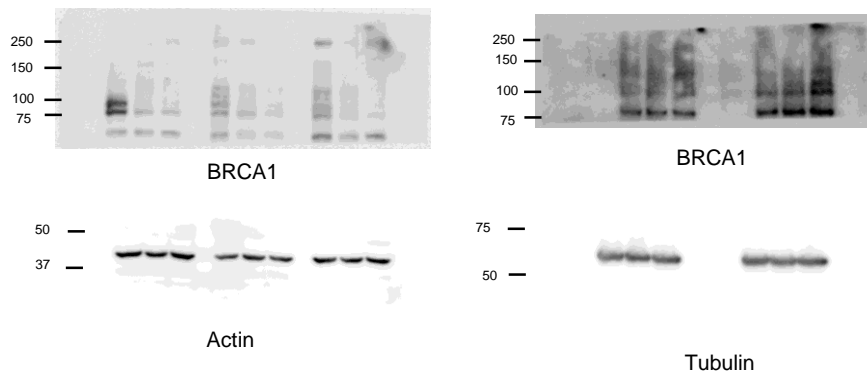

**Supplemental Fig 4G, H**

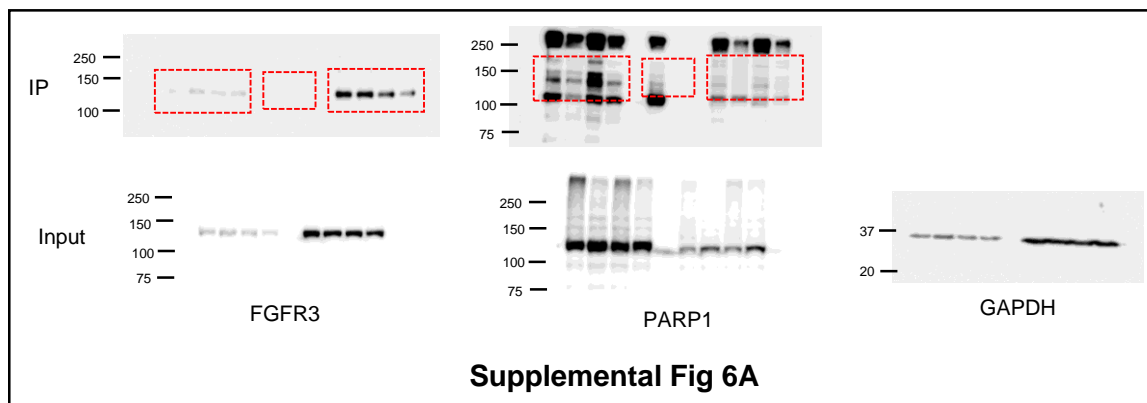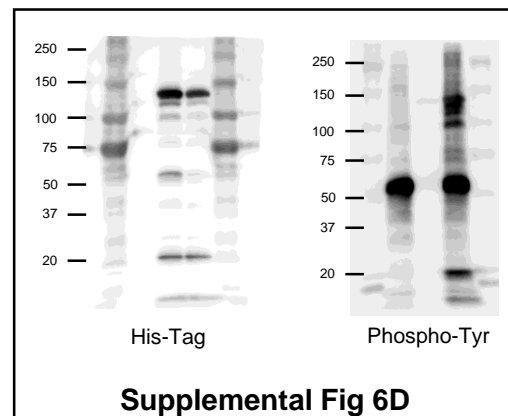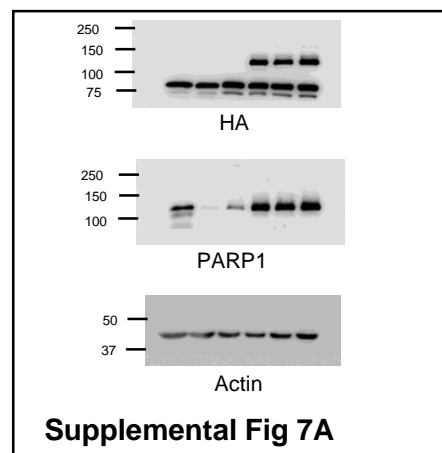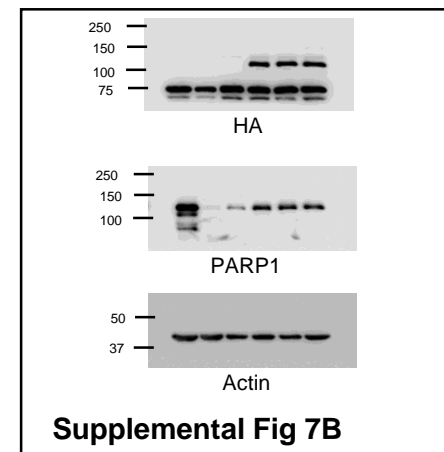

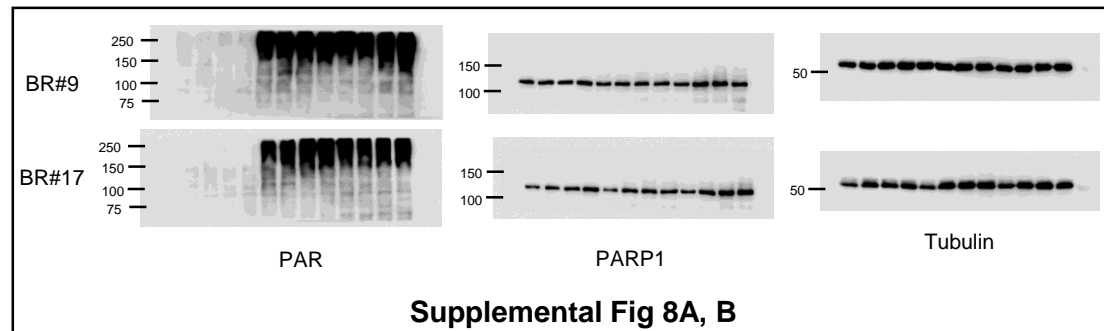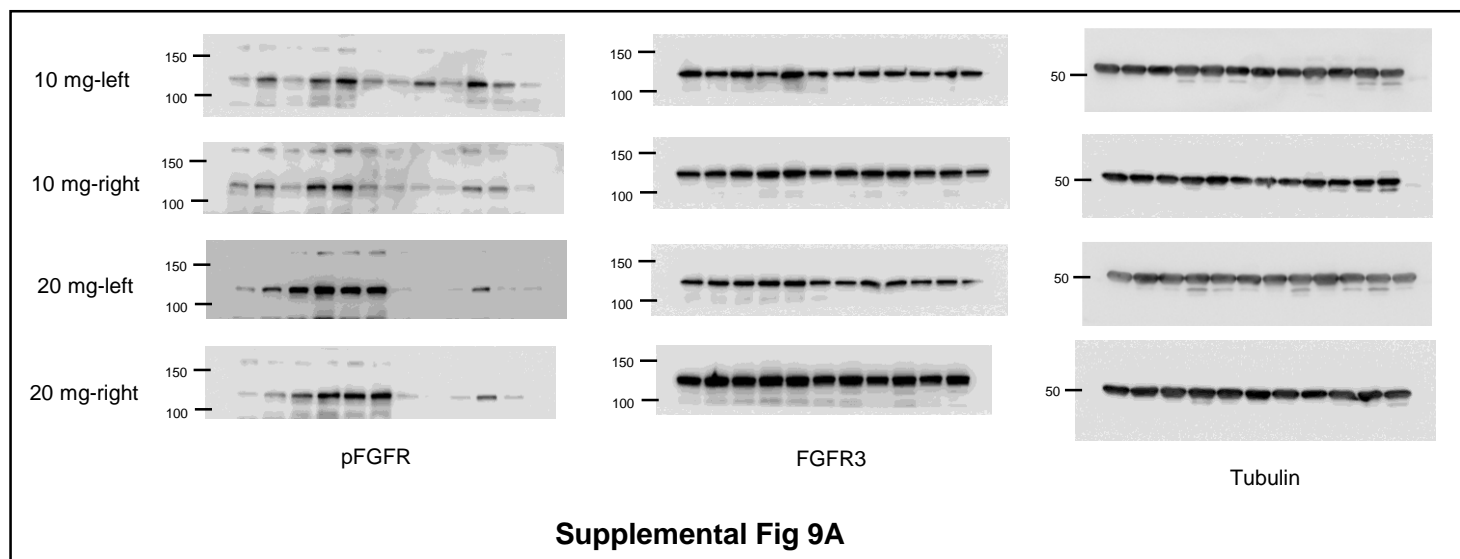

Supplement: Unedited blot and gel images [file jci-135-173757-s134.pdf]
